# Structure and evolutionary trace-assisted screening of a residue swapping the substrate ambiguity and chiral specificity in an esterase

**DOI:** 10.1016/j.csbj.2021.04.041

**Published:** 2021-04-18

**Authors:** Isabel Cea-Rama, Cristina Coscolín, Panagiotis Katsonis, Rafael Bargiela, Peter N. Golyshin, Olivier Lichtarge, Manuel Ferrer, Julia Sanz-Aparicio

**Affiliations:** a Institute of Physical Chemistry “Rocasolano”, CSIC, 28006 Madrid, Spain; b Institute of Catalysis, CSIC, 28049 Madrid, Spain; c Baylor College of Medicine, Houston, TX 77030, USA; d Centre for Environmental Biotechnology, Bangor University, LL57 2UW Bangor, UK; e School of Natural Sciences, Bangor University, LL57 2UW Bangor, UK

**Keywords:** *E*_app_, apparent enantioselectivity, ET, evolutionary trace, EA, evolutionary action, HEPES, 40 mM 4-(2-hydroxyethyl)-1-piperazineethanesulfonic acid, Ni-NTA, nickel-nitrilotriacetic acid, Crystal structure, Esterase, Evolutionary trace, Promiscuity, Protein engineering, Specificity

## Abstract

Our understanding of enzymes with high substrate ambiguity remains limited because their large active sites allow substrate docking freedom to an extent that seems incompatible with stereospecificity. One possibility is that some of these enzymes evolved a set of evolutionarily fitted sequence positions that stringently allow switching substrate ambiguity and chiral specificity. To explore this hypothesis, we targeted for mutation a serine ester hydrolase (EH_3_) that exhibits an impressive 71-substrate repertoire but is not stereospecific (*e.e.* 50%). We used structural actions and the computational evolutionary trace method to explore specificity-swapping sequence positions and hypothesized that position I244 was critical. Driven by evolutionary action analysis, this position was substituted to leucine, which together with isoleucine appears to be the amino acid most commonly present in the closest homologous sequences (max. identity, *ca*. 67.1%), and to phenylalanine, which appears in distant homologues. While the I244L mutation did not have any functional consequences, the I244F mutation allowed the esterase to maintain a remarkable 53-substrate range while gaining stereospecificity properties (*e.e.* 99.99%). These data support the possibility that some enzymes evolve sequence positions that control the substrate scope and stereospecificity. Such residues, which can be evolutionarily screened, may serve as starting points for further designing substrate-ambiguous, yet chiral-specific, enzymes that are greatly appreciated in biotechnology and synthetic chemistry.

## Highlights

• Some enzymes evolved fitted positions controlling specificity.
• These positions can be evolutionary traced.
• Engineering substrate promiscuous yet stereospecific hydrolases is feasible.

## Graphical abstract

**Figure:**
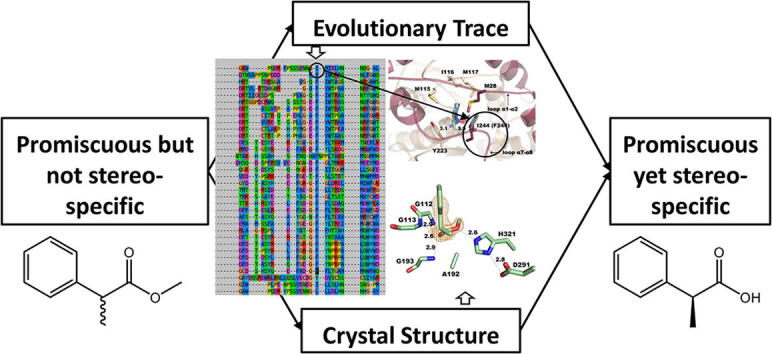

## 1 Introduction

The pivotal assets provided by the use of enzymes in industrial processes and consumer products include the following: a lower energy footprint; reduced waste production and chemical consumption; safer process conditions; and the use of renewable feedstocks. As such, replacing chemicals (including chemical catalysts) with enzymes in industrial processes or consumer products is expected to positively impact greenhouse gas emissions (reported savings from 0.3 to 990 kg CO_2_ equivalent/kg product) and global warming issues by reducing water and energy consumption (estimates: 6000 million m^3^ and 167 TWh, respectively) [1]. In particular, enzymes with broad substrate ambiguity and exact stereo-control are appreciated as candidates for developing alternative methods to conventional chemical catalysis in bench work and the pharmaceutical industry [2], [3]. However, enzymes that combine both features are rare. Indeed, most enzymes designed by nature through four billion years of evolution perform primary reactions with exquisite specificity [4]. The universe of enzymes with ambiguous specificities is also large, but the voluminous active sites selected in evolution to provide a high level of substrate docking freedom are commonly not stereospecific [5], which limits the technological potential of multi-specific (or substrate-ambiguous) enzymes. A better understanding of how substrate specificity can be modulated in such enzymes would assist engineering strategies [6] in increasing their technological impact.

Past studies have shown that enzyme specificity is influenced by the architecture (size and geometry) of their active-site cavity and by their access tunnels [7], which can evolve from an ancestral core domain or a minimal structural unit within a superfamily [8]. In general, large active sites are consistent with the very broad substrate specificity of these enzymes, whereas enzymes with smaller and occluded cavities cannot readily accommodate a larger number of substrates [7], [9]. Aside from these general trends, the presence of key substitutions in the active site and in the access tunnels [10], [11] or the positioning of water molecules [12] or anions [13] in the proximity of the active site may influence the entrance and positioning of certain substrates. In other cases, alterations in specificity were ascribed to large structural elements that are inserted, removed or rearranged in the sequence [14] or to differences in the protein dynamics [15]. Few substitutions were also found to be sufficient to modify the reaction mechanisms of enzymes, which opens the possibility to transform distinct molecules [16]. These studies exemplify that influencing and expanding the substrate specificity of enzymes is feasible. Prominent examples with remarkable substrate specificity are the human cytochrome P450 enzyme [17] and resurrected TEM-1 β-lactamases [18]. The application of multiple engineering methodologies has also demonstrated that the transformation of a nonspecific enzyme into a specific enzyme is also theoretically feasible [11], [19], [20], [21], [22], with this transformation being more effective when altering residues close to the active site or the substrate accessibility channel [23], [24].

While modulating substrate specificity in enzymes is thus feasible when examined as separate properties, introducing chiral specificity to an enzyme with prominent substrate ambiguity is challenging and has received much less attention. Few examples have been reported, such as engineered horseradish peroxidase [25], cytochrome CYP3A4 [26], peroxidase C45 [27], Michaelase [28], beta-lactamases [29] or esterase [30], which showed chiral specificity while having moderate substrate ambiguity; however, in most cases, specificity was established on the basis of a limited set of structurally similar substrates.

Here, we exploit previous comprehensive information on the substrate specificity of a large set of ester hydrolases [9] tested with close to one hundred distinct esters to identify one such enzyme, EH_3_, which has remarkable multi-specificity, with sequence positions that modulate both substrate ambiguity and chiral specificity. We focused on carboxylic ester hydrolases (EC 3.1.1), as they are among the most important biocatalysts in the field of biotechnology [31], and because of their capacity to catalyze hydrolysis with exquisite enantio-, regio-, and stereospecificity. According to their sequence, they are grouped into 19 different families with more than 1,500 available protein structures according to the lipase engineering database [32]. Through this investigation, we asked the following questions: Are there sequence positions that determine enzyme specificity? Can these positions be screened and used to produce substrate-promiscuous but chiral-specific enzymes? Answering these questions may be fundamental from a basic point of view. Thus, functional residues in enzymes tend to be highly conserved over evolution [33], [34], but to what extent certain sites impose substrate ambiguity over chiral specificity and, conversely, their conservation through evolution are not known. This is of special significance given that genome-scale model simulations and laboratory evolution experiments have shown that few mutations shift enzyme substrate turnover rates toward new substrates, thus shaping microbial adaptation to novel growth substrates [35]. From a technological point of view, answering these questions will also have implications for fine-tuning enzyme specificity. For the purpose of this study, we herein explore the evolutionary importance of sequence positions that possibly have functional roles in the chiral specificity of substrate ambiguous esterase through the application of a software program called Evolutionary Trace [36], [37] and structure-assisted and experimental validations. We would like to highlight that previous work on evolutionary traces [38] focused on altering the substrate specificity for a few substrates, and to the best of our knowledge, their application to modulate enzyme specificity in combination with substrate promiscuity has not yet been reported.

## 2 Materials and methods

### 2.1 Enzyme source, production and purification

The vector pBXNH3 and the host *Escherichia coli* MC1061 were the sources of His_6_-tagged EH_3_ (GenBank acc. nr. KY483645), a serine ester hydrolase isolated from the metagenomic DNA of microbial communities inhabiting the chronically polluted seashore area of Milazzo Harbor in Sicily [9]. The soluble His-tagged protein was produced and purified at 4 °C after binding to a Ni-NTA His-Bind resin (from Merck Life Science S.L.U., Madrid, Spain) as described previously [39]. The purity was assessed as >98% using SDS-PAGE analysis in a Mini PROTEAN electrophoresis system (Bio-Rad, Madrid, Spain). Purified protein was stored at −86 °C until use at a concentration of 10 mg ml^−1^ in 40 mM 4-(2-hydroxyethyl)-1-piperazineethanesulfonic acid (HEPES) buffer (pH 7.0). A total of approximately 20 mg of total purified recombinant protein was obtained from a 1-liter culture.

### 2.2 Source of chemicals

The source or brand for each of the esters [purity ≥ 99%] used in this study has been described previously [9]. Methyl-(*R*)-2-phenylpropanoate and methyl-(*S*)-2-phenylpropanoate [purity ≥ 99%] were purchased from Combi-Blocks (San Diego, CA, USA). HEPES [purity ≥ 99%] was purchased from Fisher Bioreagent (Ottawa, ON, USA). All other chemicals [with the highest purity available] were purchased from Merck Life Science S.L.U., Madrid, Spain) and Sigma-Aldrich Química S.A. Madrid (Spain).

### 2.3 Crystallization and X-ray structure determination of EH_3_ complexed with methyl-(R/S)-2-phenylpropanoate

The crystallization conditions reported for the native protein were optimized by adjusting the protein and precipitant concentrations. The best crystals were grown by using 1 µl of EH_3S192A_ (20–60 mg ml^−1^ in 40 mM HEPES (pH 7) and 100 mM NaCl) and 0.5 µl of precipitant solution (28–29% PEG3000, 0.1 M Bis-tris (pH 6.5), and 0.2 M MgCl_2_∙6H_2_O). The complexes were obtained by soaking thin plate-shaped crystals of EH_3S192A_ in mother liquor supplemented with 10–20 mM methyl-(*S*/*R*)-2-phenylpropanoate for 1–3 h. For data collection, crystals were transferred to cryoprotectant solutions consisting of mother liquor plus 20–23% (v/v) glycerol before being cooled in liquid nitrogen. Diffraction data were collected using synchrotron radiation on the XALOC beamline at ALBA (Cerdanyola del Vallés, Spain). Diffraction images were processed with XDS [40] and merged using AIMLESS [41] from the CCP4 package [42]. Both crystals were indexed in the C2 space group, with two molecules in the asymmetric unit and 40% solvent content within the unit cell. The data collection statistics are given in Table S1.

The structure of the complex was solved by difference Fourier synthesis using the coordinates of the EH_3_ native crystals (PDB ID: 6SXP). Crystallographic refinement was performed using the program REFMAC [43] within the CCP4 suite with local noncrystallographic symmetry (NCS). The free R-factor was calculated using a subset of 5% randomly selected structure-factor amplitudes that were excluded from the automated refinement. At the later stages, ligands were manually built into the electron density maps with Coot [44], and water molecules were included in the model, which, when combined with more rounds of restrained refinement, reached the R factors listed in Table S1. For methyl-(*R*/*S*)-2-phenylpropanoate, which is not present in the Protein Data Bank, a model was built using MacPyMOLX11Hybrid (the PyMOL Molecular Graphics System, Version 2.0, Schrödinger, LLC). The model was used to automatically generate coordinates and molecular topologies with eLBOW [45], which is suitable for REFMAC refinement. The figures were generated with PyMOL. The crystallographic statistics of EH3_S192A_ complexed with methyl-(*R*/*S*)-2-phenylpropanoate are listed in Table S1.

### 2.4 Site-directed mutagenesis

Mutagenic PCR was performed using the QuikChange Lightning Multi Site-Directed Mutagenesis Kit (Agilent Technologies, Cheadle, UK), as described previously [22]. The forward primers used to generate the EH_3I244L_ and EH_3I244F_ variants were as follows: 5′-GCGAAAACAATGGCCTCATGATTGAACTGCATAAC-3′ and 5′-GCGAAAACAATGGCTTCATGATTGAACTGCATAAC-3′, respectively. The pBXNH3 plasmid containing EH_3_ DNA [9] was used as a template to perform mutagenic PCR.

### 2.5 Hydrolytic activity assessment

Ester hydrolysis was assayed using a pH indicator assay in 384-well plates at 30 °C and pH 8.0 in a Synergy HT Multi-Mode Microplate Reader in continuous mode at 550 nm over 24 h. Conditions were as detailed previously [39]. For *K_m_* determination, [protein]: 4.5 μg ml^−1^; [ester]: 0–100 mM; reaction volume: 44 μl; T: 30 °C; and pH: 8.0. For *k*_cat_ determination, [protein]: 0–270 μg ml^−1^; [ester]: 50 mM; reaction volume: 44 μl; T: 30 °C; and pH: 8.0.

The effect of pH on the activity was determined in 50 mM Britton and Robinson buffer at pH 4.0–12.0, following the production of 4-nitrophenol from the hydrolysis of 4-nitrophenyl-propionate (*p*NPC_3_: 0.8 mM) at 348 nm (ε = 4147 M^−1^ cm^−1^) over 5 min and determining the absorbance per minute from the slopes generated [22]. Reactions, performed at 30 °C, each contained 2 μg of protein in a total volume of 200 μl. Similar assay conditions were used to assay the effects of temperature on esterase hydrolysis of *p*NPC_3_, but in this case, reactions were performed in 50 mM Britton and Robinson buffer pH 8.0.

All values, in triplicate, were corrected for nonenzymatic transformation. The absence of activity was defined as at least a twofold background signal as described [39].

### 2.6 Hydrolysis of methyl-(R/S)-2-phenylpropanoate and gas chromatography (GC) analysis

Prior to the use of the racemic mixture, the continuous hydrolysis of separate methyl (*R*)-2-phenylpropanoate and methyl (*S*)-2-phenylpropanoate was performed. Briefly, 2 µl of each enantiomer (from a stock solution of 200 mg ml^−1^ in acetonitrile) was added to 96 µl of 5 mM 4-(2-hydroxyethyl)-1-piperazinepropanesulfonic acid (EPPS) buffer (pH 8.0) containing 0.9 mM Phenol Red (Merck Life Science S.L.U., Madrid, Spain). Then, 2 µl of enzyme solution (from a stock solution of 1.0 mg ml^−1^ in 40 mM HEPES buffer, pH 7.0) was added, and the progress of the reaction at 30 °C was followed continuously at 590 nm. These reaction conditions were set up to evaluate the chiral specificity using a racemic ester of methyl (*R*/*S*)-2-phenylpropanoate. After 60 min, reactions with racemic mixtures were stopped by adding 1800 µl of HPLC-grade methanol, and the reaction products were analyzed by GC through a GC-Column CP-Chirasil-Dex CB (25 m length, 0.25 µm internal diameter, 0.25 μm film) (Agilent J&W GC Columns), as previously described [22].

### 2.7 Circular dichroism to estimate the thermal denaturation of EH_3_

Circular dichroism (CD) spectra were acquired between 190 and 270 nm with a Jasco J-720 spectropolarimeter equipped with a Peltier temperature controller, employing a 0.1-mm cell at 25 °C. Spectra were analyzed, and denaturation temperature (T_d_) values were determined at 220 nm between 10 and 85 °C at a rate of 30 °C per hour in 50 mM Britton and Robinson buffer at pH 8.5. A protein concentration of 1.0 mg ml^−1^ was used. T_d_ (and standard deviation of the linear fit) was calculated by fitting the ellipticity (mdeg) at 220 nm at each of the different temperatures using a 5-parameter sigmoid fit with SigmaPlot 13.0.

### 2.8 Cavity volume and solvent-accessible surface area (SASA) calculation

The relative solvent-accessible surface area (SASA) of the active site, computed as a (dimensionless) percentage of the ligand SASA in solution, was obtained using the GetArea web server [46]. Note that the relative SASA of the catalytic triad (derived from the GetArea server) adopts values of 0–100. The volume of the active site cavity was computed with fpocket [47], which is a very fast open-source protein pocket (cavity) detection algorithm based on Voronoi tessellation. fpocket includes two other programs (dpocket and tpocket) that allow the extraction of pocket descriptors and the testing of owned scoring functions, respectively.

### 2.9 Evolutionary trace and evolutionary action computations

The evolutionary importance of sequence positions was estimated using the *Evolutionary Trace (ET) method* [36], [37], which is available at http://lichtargelab.org/software/ETserver. ET scores the functional importance of protein sequence positions by quantifying the correlation of variations in homologous proteins with the phylogenetic divergence of the sequences. Residue variations associated with large phylogenetic distances indicate important residues, and vice versa. The ET output is given as a top-ranked score (on the scale of 0 for the most important to 100 for the least important residues), which indicates the percentage of protein residues that were found to be more important than the residue of interest.

The functional impact of the potential amino acid substitutions was estimated using the *Evolutionary Action (EA) method* [48], which is available at http://eaction.lichtargelab.org/. EA estimates the evolutionary impact of sequence changes through a simple model of protein evolution that accounts for the evolutionary importance of the residue (ET method) and for the similarity of the substitution. The similarity of the substitution is quantified through substitution odds that are specific to the evolutionary importance, secondary structure, and solvent accessibility of each residue. The outcome is a rank score that indicates the percentage of all potential amino acid changes in the protein that are predicted to have less impact than the substitution of interest. Therefore, EA is given on a scale from 0 (fully neutral) to 100 (fully deleterious).

Both ET and EA require to input an alignment of homologous sequences. We generated the input alignment using the default parameters of the ET server (UniRef90, 20% minimum sequence identity, 0.5 minimum fractional length to query), which resulted in 410 homologous sequences.

## 3 Results and discussion

### 3.1 Biochemical and substrate specificity characteristics of EH_3_

EH_3_ was identified in a recent study as the third most substrate-ambiguous ester hydrolase out of 145 tested enzymes [9]. This enzyme, which belongs to family IV of the Arpigny and Jaeger classification [31], originated from an uncultured bacterium of the genus *Hyphomonas* (phylum *Proteobacteria*), a highly versatile group of halophiles in terms of their ability to successfully grow in a variety of environmental conditions and capable of mineralizing a high number of pollutants [49]; this may be in agreement with the fact that this enzyme was isolated from a chronically polluted seashore area [9].

**Fig. 1:**
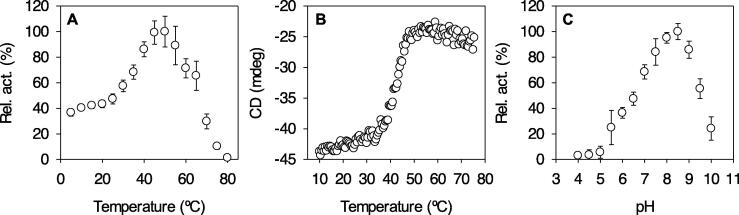
Optimal parameters for the activity and stability of purified EH_3_. (A) Temperature profile determined as follows: protein, 2 μg; [*p*-nitrophenyl propionate (*p*NPC_3_)], 0.8 mM; pH, 50 mM Britton and Robinson buffer pH 8.0; T, 5–80 °C; reaction volume, 200 μl. (B) The thermal denaturation curve of EH_3_ at pH 7.0 was measured by ellipticity changes at 220 nm and obtained at different temperatures. (C) The pH profile was determined as follows: protein, 2 μg; [*p*NPC_3_], 0.8 mM; T, 30 °C; pH, 50 mM Britton and Robinson buffer from 4.0 to 10.0; reaction volume, 200 μl. Graphics were created with SigmaPlot version 14.0. The data are not fitted to any model.

**Fig. 2:**
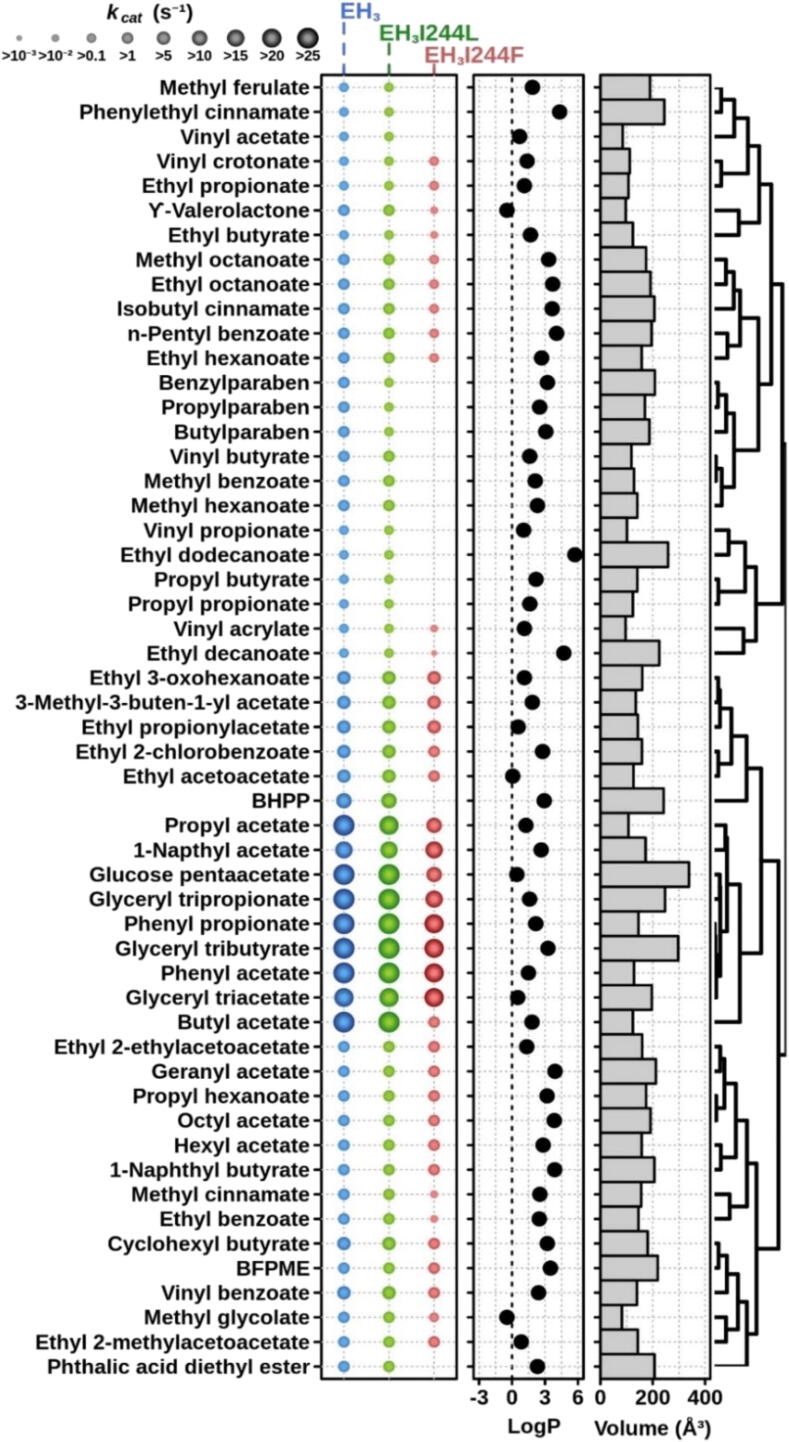
Non-chiral substrate specificity. The *k_cat_* (s^−1^) values of the EH_3_, EH_3I244L_ and EH_3I244F_ variants were measured for 53 non-chiral carboxylic esters found to be hydrolyzed by any of the enzyme variants. The substrates, with the hydrophobicity (log P) and volume (Å^3^) indicated (details in Table S2), are ranked based on hierarchical clustering according to substrate similarity profiles. For *k_cat_* determination, calculated on a continuous pH indicator assay, the conditions were as follows: [enzyme], 0–270 µg ml^−1^; [ester], 50 mM to ensure substrate saturation; reaction volume, 44 µl; T, 30 °C; and pH, 8.0. Abbreviations are as follows: BFPME: benzoic acid, 4-formyl-, phenylmethyl ester; BHPP: benzyl (*R*)-2-hydroxy-3-phenylpropionate. LogP values and molecular volume of each ester were calculated using ACD/ChemSketch 2015.2.5 and Molinspiration software, respectively. For raw data and details, see Table S2.

**Fig. 3:**
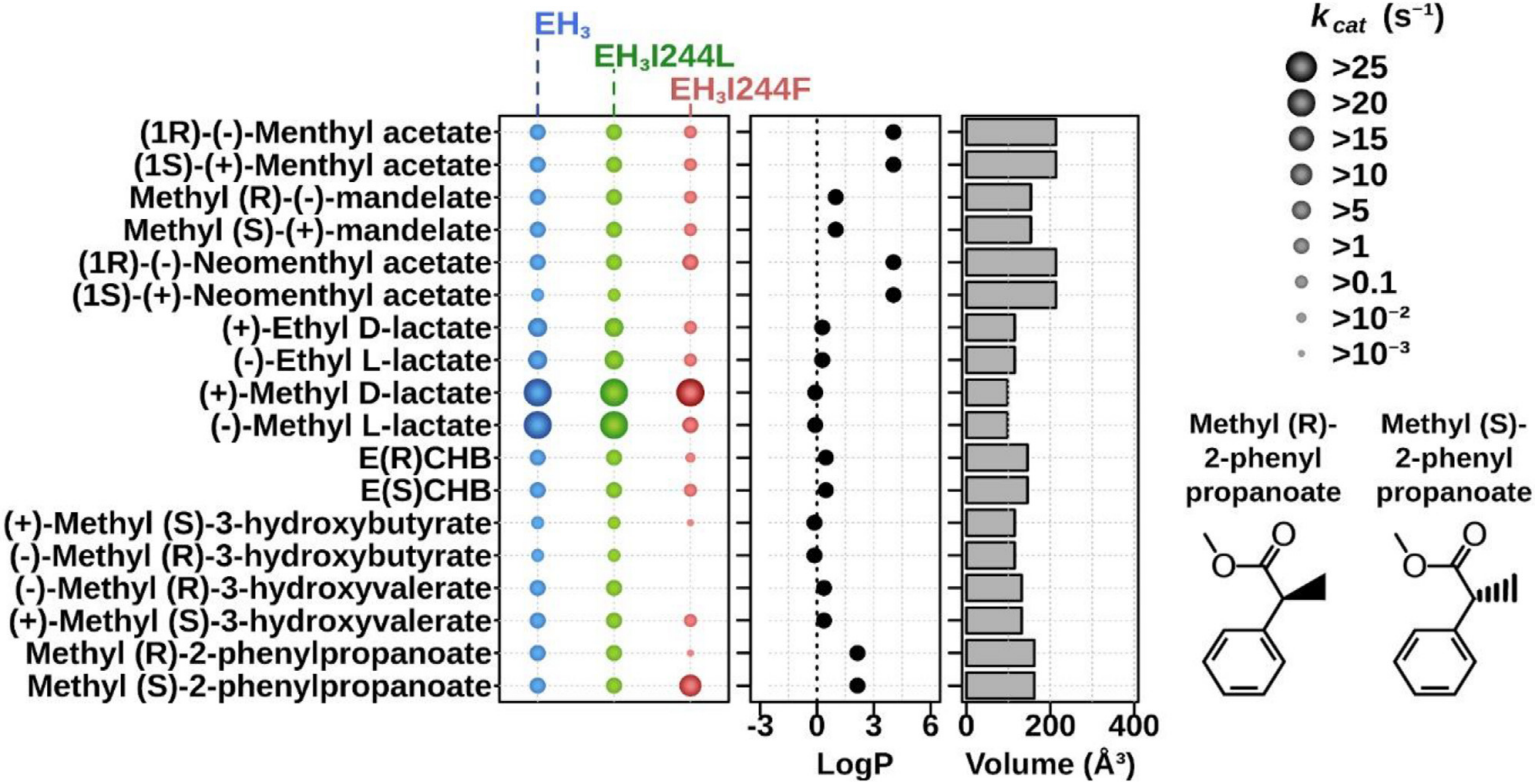
Chiral substrate specificity. The *k_cat_* (s^−1^) values of the EH_3_, EH_3I244L_ and EH_3I244F_ variants measured for 18 chiral carboxylic esters found to be hydrolyzed by any of the enzyme variants. Abbreviations are as follows: E(R)CHB, ethyl (*R*)-4-chloro-3-hydroxybutyrate; E(S)CHB, ethyl (*S*)-4-chloro-3-hydroxybutyrate. Figure preparation and experimental details are shown in Fig. 2. The structures of methyl-(*R*)-2-phenylpropanoate and methyl-(*S*)-2-phenylpropanoate used for soaking and investigation of chiral specificity are shown. LogP values and the molecular volume of each ester were calculated using ACD/ChemSketch 2015.2.5 and Molinspiration software, respectively. For raw data and details, see Table S2.

EH_3_ did show maximal activity at 50 °C, retaining more than 80% of the maximum activity at 40–55 °C (Fig. 1A), suggesting that it is moderately thermostable. This was confirmed by circular dichroism analysis, which revealed a denaturing temperature of 45.90 ± 0.43 °C (Fig. 1B). Its optimal pH for activity is 8.5 (Fig. 1C). Its voluminous (volume of the active site cavity: 1718.02 Å^3^) but low exposed (solvent accessible surface area (SASA): 6.03 over 100 dimensionless percentage) active site allows hydrolysis of a broad range of 71 structurally and chemically diverse esters, including non-chiral (Fig. 2) and chiral (Fig. 3) esters. Such topology, namely, active site cavities with large volume but low exposition to the surface, has been found to be beneficial for retaining a higher number of substrates in specific catalytic binding interactions and thus for promoting substrate promiscuity [9]. However, it is not stereospecific according to the quick apparent enantioselectivity (*E_app_*) method [50], in which the ratios between the *k*_cat_/*K*_m_ of the preferred chiral ester and the nonpreferred chiral ester (from *ca*. 1.02 to 6.93; Table 1) were calculated when tested separately.

**Table 1:** *E_app_* values for the hydrolysis of separate pairs of enantiomers.

Chiral pair (*R*/*S*)	*E_app_*: (*k*_cat_/*K*_m_ preferred)/(*k*_cat_/*K*_m_ nonpreferred)^1^		
	EH_3_	EH_3I244L_	EH_3I244F_
Menthyl acetate	1.71 ± 0.25 (*S*)	1.50 ± 0.39 (*S*)	6.40 ± 0.37 (*S*)
Methyl mandelate	1.53 ± 0.24 (*S*)	1.93 ± 0.15 (*S*)	3.24 ± 0.04 (*S*)
Neomenthyl acetate	6.93 ± 0.35 (*R*)	6.88 ± 0.14 (*R*)	100% specific (*R*)
Methyl lactate	2.35 ± 0.11 (*R*)	2.49 ± 0.03 (*R*)	226.5 ± 4.5 (*R*)
Ethyl lactate	1.74 ± 0.16 (*R*)	1.76 ± 0.21 (*R*)	9.03 ± 0.91 (*R*)
Ethyl-4-chloro-3-hydroxybutyrate	1.59 ± 0.18 (*R*)	1.39 ± 0.05 (*R*)	6.22 ± 0.28 (*R*)
Methyl-3-hydroxybutyrate	1.33 ± 0.34 (*R*)	1.27 ± 0.16 (*R*)	100% specific (*R*)
Methyl-3-hydroxyvalerate	1.02 ± 0.10 (*R*)	1.09 ± 0.14 (*R*)	100% specific (*R*)
Methyl-2-phenylpropanoate	2.21 ± 0.08 (*S*)	2.16 ± 0.05 (*S*)	56300 ± 42 (*S*)

1 Calculated by following the hydrolysis of separate enantiomers in a continuous high-throughput pH indicator assay (see Materials and methods).

### 3.2 Insights into the structural basis of EH_3_ substrate ambiguity

**Fig. 4:**
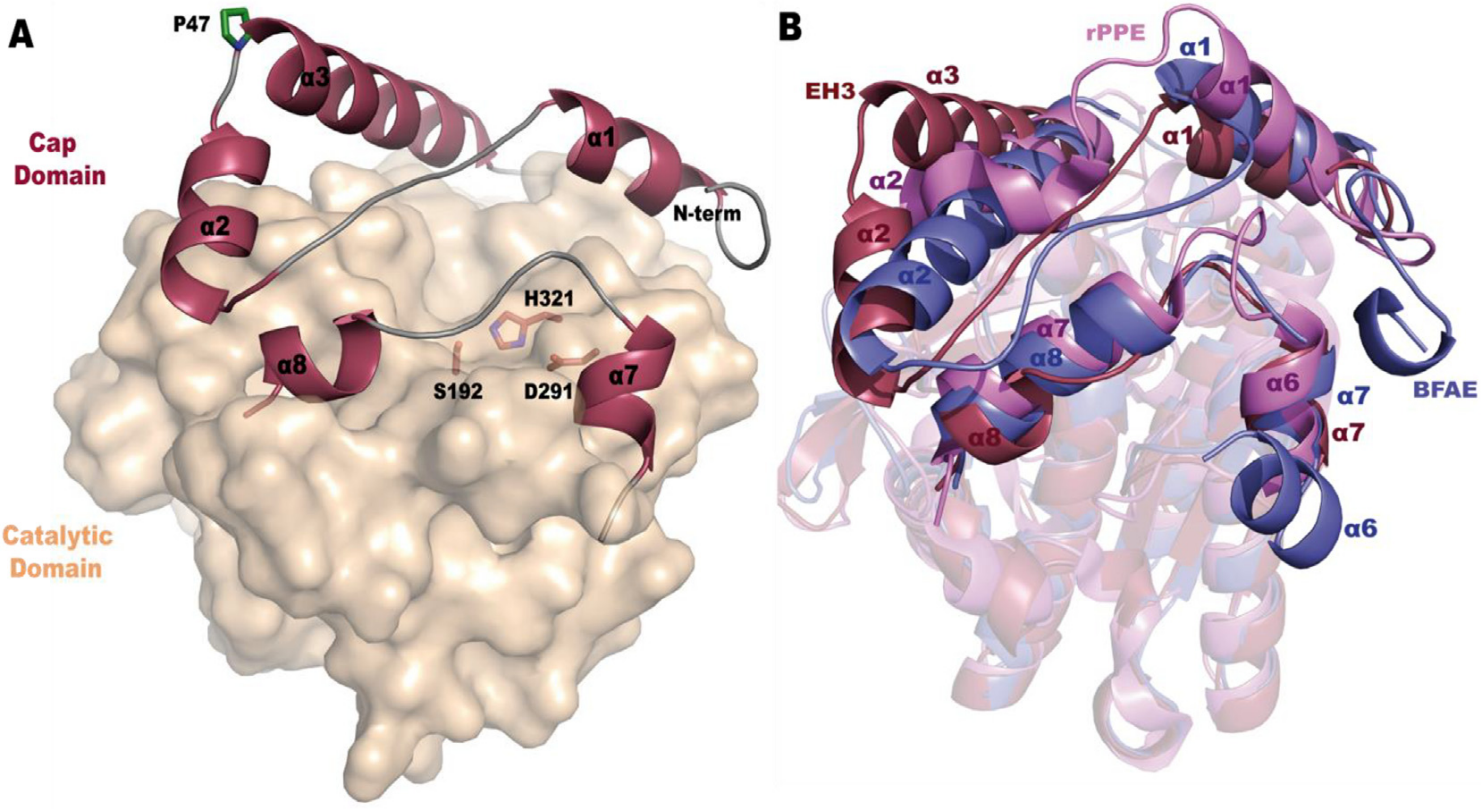
Crystal structure of EH_3_. (A) Molecular surface of the catalytic domain (wheat) with the α-helices making up the cap domain depicted as a cartoon (plum); for secondary structure numbering, see Fig. S1. The catalytic triad is shown as sticks (orange). The region comprising α1- α2 is highly flexible, and P47 acts as a hinge (green sticks). (B) Superimposition of the EH_3_ subunit (plum) and its homologues, BFAE (slate, PDB ID: 1JKM) and rPPE_S159A/W187H_ (violet, PDB ID: 4OB6). The cap domain presents the largest differences that configure markedly divergent active sites. The folding characteristics of Est22 and Est25 are most similar to those of EH_3_ and BFAE, respectively, and have been omitted for clarity. (For interpretation of the references to color in this figure legend, the reader is referred to the web version of this article.)

As previously reported by us [39], the crystal structure of EH_3_ showed that it is folded into two different domains: an α/β-hydrolase catalytic domain housing the catalytic triad (S192, A291, and H321) and a cap domain located on top and preventing the entrance of substrates into the active site (Fig. 4A). The polypeptide chain is folded into a total of eleven α-helices and eight β-sheets; five of the α-helices compose the cap domain, three at the N-terminus (α1, α2, α3) and two more (α7 and α8) after strand β6 from the central sheet (Fig. S1). The analysis of the B factor values revealed that the cap region comprising α1- α2 is highly flexible, with the loop linking both α-helices being partially disordered in the native structure but becoming more ordered upon substrate binding.

To disclose the molecular basis behind the substrate ambiguity, we compared the EH_3_ structure with other reported esterases. As expected, this highly flexible cap is the most variable region among homologues. Analysis of EH_3_ folding using the *DALI* server shows that its closest homologue is Est22, which was isolated from environmental samples, with 64% identity and an RMSD of 0.9 Å from 336 Cα atoms [51] (PDB ID: 5HC0). Other homologues are Est25 from environmental samples (RMSD of 1.8 Å from 323 Cα atoms, PDB ID: 4J7A [52]), Brefeldin A (BFAE) from *Bacillus subtilis* (RMSD of 2.0 Å from 323 C α atoms, PDB ID: 1JKM [53]) and the carboxylesterase rPPE from *Pseudomonas putida* (RMSD of 2.0 Å from 297C α atoms, PDB ID: 4OB6 [54]), and these three proteins are 20–40% identical to EH_3_. They all belong to the hormone-sensitive lipase (HSL) family or family IV [31]. This HSL family presents a very conserved folding at the core α/β domain, with the largest differences at the cap domain that, consequently, must be mostly responsible for their different functionalities (Fig. 4B). First, the loop connecting helices α1 and α2 is very short in rPPE, and as a result, the active site cavity of this protein is reduced, allowing relatively small substrates to enter. Moreover, the EH_3_ and Est22 α2 and α3 helices are fused into a unique long α -helix in BFAE and Est25. Although this arrangement in two separate, more mobile helices is shared with Est22, EH_3_ presents a proline residue at the beginning of α3 (P47, but this residue is a glutamate in Est22), which could act as a hinge to increase the mobility of the EH_3_ α1- α2 moiety (Fig. 4A). This feature might be an additional mechanism that adapts the topology of the EH_3_ active site to a higher variety of substrates and explains its observed substrate promiscuity. Furthermore, the shorter α8 in EH_3_ makes a longer α7- α8 loop and a wider catalytic site, probably also contributing to the superior substrate promiscuity of EH_3_. Moreover, as the homologous HSL enzyme, EH_3_ is a homodimer where both subunits are related by a twofold symmetry axis (Fig. S2, Table S3).

To conclude, EH_3_ may be considered a moderately thermostable serine ester hydrolase with prominent substrate ambiguity but is not stereospecific. This is the result of its novel capacity to adapt the topology of the large but occluded active site to a high variety of substrates.

### 3.3 Evolutionary screening of specificity swapping positions

**Fig. 5:**
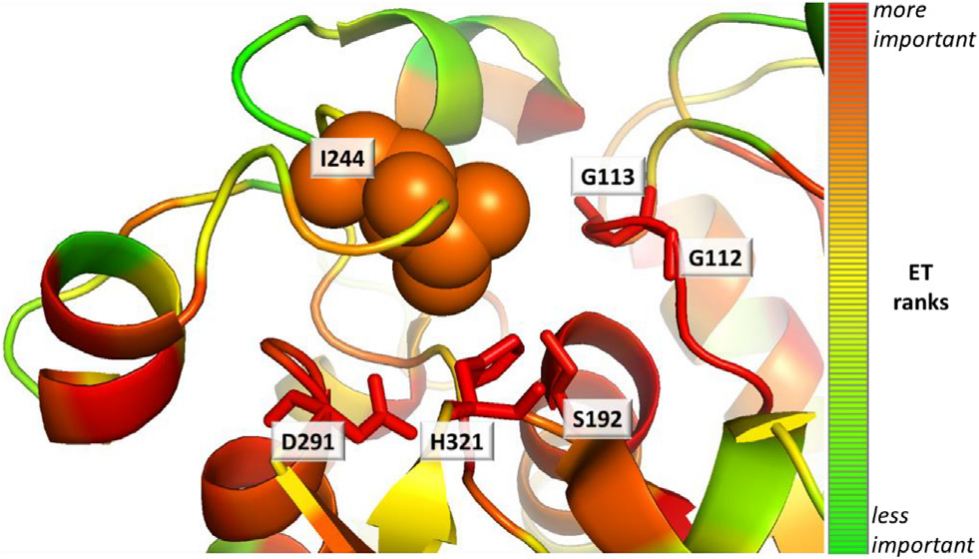
Evolutionary trace ranks for the EH_3_ protein. The analysis used 410 homologous sequences of EH_3_ with sequence identity as low as 20%. The ET ranks are represented on the structure with a color scale (the most important residues are red, and the least important residues are green). While the catalytic residues were ranked within the top most important residues (S192 was 3%, D291 was 2%, and H321 was 1%), residue I244 was ranked in the top 12%, and it was the most important residue of loop 240–249 in contact with the catalytic residues. The figure was generated using the PDB structure 6SXP, PyMOL (version 1.8), ET (with the position-specific option), and the PyMOL ET viewer [55]. (For interpretation of the references to color in this figure legend, the reader is referred to the web version of this article.)

To explore the functional roles of sequence positions, we used the Evolutionary Trace (ET) method [36], [37]. In previous work [38], ET identified few key sequence positions that were able to alter the substrate specificity of homologous proteins; therefore, we hypothesized that ET would also be able to identify positions that modulate enzyme specificity in combination with substrate promiscuity. According to the ET ranks for the EH_3_ protein (shown in Table S4), position 244 was ranked within the top 12% of residues, and it is the most important residue of the loop formed by residues 240–249 (loop α7- α8 at the cap, Fig. 4A), which are in contact with the catalytic triad (Fig. 5).

Leucine and isoleucine are amino acids that are most commonly present (*ca*. 70% of the closest homologous sequences) at position 244, as shown in the alignment, while other amino acids, such as tryptophan and valine, appear less frequently and mostly in distant homologs (Table 2). This was also confirmed when we used BLAST to search for the EH_3_ sequence in the nonredundant (nr) [56], UniProt [57], and Marref, MarDB and MarCat [58] databases. We were able to report up to 10,000 alignment hits with a minimum query coverage of 50% and an e-value cutoff of 1e^-10^, ensuring in all cases the correct alignment of the three residues forming the catalytic triad (S192, D291, and H321), the two residues (G112 and G113) forming the so-called oxyanion hole-stabilizing substrates, and the residue (P47) acting as a hinge that allows mobility of the cap domain to control substrate access to the catalytic site. Above an identity of 50%, all homologues contain either isoleucine (top homologue WP_156780860.1; identify, 67%; e-value, 3e^-176^) or leucine (top homologue AKJ87259.1; identify, 66%; e-value, 7e^-168^), while TNF86759.1 (identify 67%; e-value 3e^-169^) contains a methionine, and E3QWZ9 (identify 35%; e-value 2e^-45^) contains a phenylalanine (Table 2). Variability at this position was only found to a higher extent at identities below 39.38% and e-values above 2.62 × 10^-69^ (Table 2).

**Table 2:** Frequency of amino acids at position I244 (following EH_3_ numeration) in EH_3_-homologous proteins as detected by ET analysis and the top homologs.

AA at 244^1^	Frequency (%)1	Top homologs		
		Accession number	Identity (%)	E-value
L	63.08	AKJ87259.1^2^	66.00	7.00 × 10^-168^
W	19.56	MBE82488.1^3^	32.49	1.32 × 10^-30^
I	8.07	WP_156780860.1^2^	67.00	3.00 × 10^-176^
V	3.18	HAY66678.1^3^	40.78	8.13 × 10^-66^
N	2.20	WP_042512518.1_MMP04251492^3^	31.35	2.80 × 10^-23^
T	0.98	GCA_002427755.1^3^	32.18	5.84 × 10^-31^
F	0.73	E3QWZ9^1^	35.00	2.00 × 10^-45^
A	0.49	WP_073577520.1^3^	33.42	5.14 × 10^-53^
H	0.49	POP51947.1_MMP08281192^3^	31.23	1.34 × 10^-21^
M	0.49	TNF86759.1^2^	67.00	3.00 × 10^-169^
G	0.24	MMP491463_308377^3^	38.18	2.47 × 10^-56^
P	0.24	WP_071722916.1_MMP05231544^3^	30.15	6.93 × 10^-23^
S	0.24	GCA_002389675.1^3^	32.05	4.22 × 10^-27^

^1^As a default, the server uses the UniRef90 database. This database was created after filtering out sequences so that it does not contain duplicates or similar sequences (higher sequence identity than 90%) among its members. This makes it a good source to find “more representative” full-length sequences (fragments and short sequences were removed) of the protein family evolution and indeed results in better ET accuracy than using more sequences from other databases. The BLAST option for sequence identity was 20% (min.) to 95% (max.). The e-value cutoff was 0.05, and up to 500 sequences were selected (above this number of representative sequences, the ET scores no longer improved). Based on these results, the different amino acids (AAs) found at position 244 (following EH_3_ numbering) are given.

^2^nr database (https://blast.ncbi.nlm.nih.gov).

^3^Other databases: UniProt (https://www.uniprot.org/) and MAR (https://mmp.sfb.uit.no/blast/).

### 3.4 Crystal structure of the substrate-bound form of EH_3_ to determine the functional role of I244

**Fig. 6:**
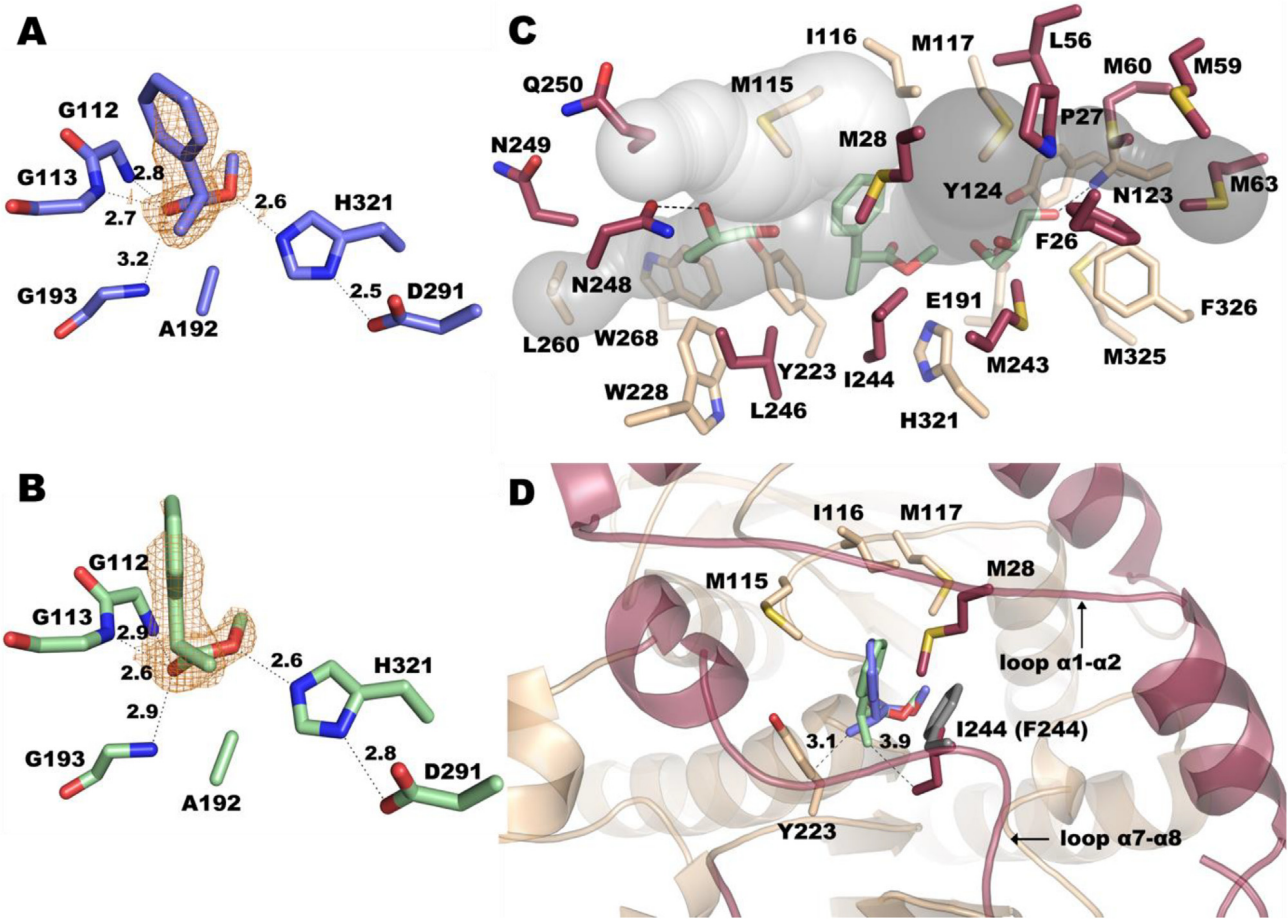
Active site of EH_3_. (A) Methyl (2*R*)-2-phenylpropanoate and (B) methyl (2*S*)-2-phenylpropanoate bound at the catalytic site of EH_3S192A_, showing the 2Fo-Fc electron density maps contoured at 0.9 and 0.8 σ in orange. (C) Active site channels of EH_3S192A_, as calculated by CAVER [59], with bound methyl (2*S*)-2-phenylpropanoate and two glycerol molecules. The residues surrounding each cavity are shown. (D) Nearest environment and conserved binding mode of methyl (2*R*)-2-phenylpropanoate (slate) and methyl (2*S*)-2-phenylpropanoate (pale green) in the complexes; the closest distance from each substrate to the EH_3_ residue is shown. The putative position of the modeled I244F mutant is shown as gray sticks. Panels C and D show the same color code as Fig. 4A. (For interpretation of the references to color in this figure legend, the reader is referred to the web version of this article.)

Our evolutionary trace analysis suggested that a single residue at position 244 potentially had a functionally important role in EH_3_. Soaking of inactivated EH_3S192A_ crystals in a solution containing either methyl-(*R*)-2-phenylpropanoate or methyl-(*S*)-2-phenylpropanoate was performed in this study to further investigate whether I244, or other amino acid residue(s) if any, is close to the substrate’s stereo-center and plays a functional role in specificity, as suggested by ET analysis. This chiral ester was selected as a model because it is structurally similar to ibuprofen-like esters that are of great industrial relevance, and the wild-type enzyme showed a lack of specificity for these chiral esters based on the *E_app_* value (Table 1). The crystal structures of these complexes were solved using the coordinates of wild-type EH_3_ (PDB ID: 6SXP). The final models were refined to crystallographic R-factors of 0.2100 and 0.1919 and R-free values of 0.2403 and 0.2276 with resolutions of 2.27 and 2.06 Å (PDB IDs: 6SYA and 6SXY), respectively. Both crystals present two molecules in the asymmetric unit forming the dimer and one ligand bound per catalytic site (Fig. 6A and 6B).

The catalytic triad of EH_3_ is formed by S192, D291 and H321. There are three conserved motifs in its sequence, ^110^HGGG^113^ (containing two of the glycines involved in the oxyanion hole), the pentapeptide ^190^GXSXG^194^ (housing the nucleophile serine and a third glycine) and ^291^DPLRDEG^297^ (including D291). The substrates are bound by polar interactions of its free carboxylate oxygen with the three glycines forming the oxyanion hole and hydrogen bonds of the ester oxygen to H321 from the catalytic triad (Fig. 6A and 6B). Structural superimposition of the wild-type coordinates with the complexes presented here shows no structural changes in the EH_3_ active site upon complex formation, and both complexes maintain high B factor values for the cap domain. As we previously described, the EH_3_ active site cavity possesses three long channels giving access to catalytic S192, an acyl binding site (approximately 11.2 Å), an alcohol binding site (10.9 Å) and a third channel that can possibly allocate substrates with branched acyls (Fig. 6C). In the complex reported here, the acyl channel is partially occupied by phenyl/methyl rings, whereas the alcohol binding channel is allocated to a small aliphatic group (methyl). Chain B from both complexes also accommodates two molecules of glycerol coming from the cryoprotectant, one at the acyl moiety and the other at the alcohol site. As seen in Fig. 6C, all three channels are shaped by mostly hydrophobic residues from the cap and the catalytic domains that, in principle, would not present specific interactions with the substrates, explaining the EH_3_ promiscuity and absence of stereospecificity. Thus, residues M115, Y223, W228, L246, I244 and L260 protrude at the acyl channel, making a mostly hydrophobic tunnel where only N248 seems able to make polar interactions with the trapped glycerol molecule. In the alcohol channel, hydrophobic residues F26, L56, M59, M60 and M63 emerge, among others, and only two polar residues, N123 and E191, form hydrogen bonds with the glycerol trapped within this channel.

A close inspection of the substrate complexes reveals the main features of the binding modes of both isomers (Fig. 6D). Keeping the same polar interactions at the carboxylate ester moiety shown in Fig. 6A and 6B, the orientation of their bulky phenyl ring is slightly adjusted in a hydrophobic pocket surrounded by M115-I116-M117 from the catalytic domain and M28 from the cap α1- α2 loop. The position of the aromatic ring is tilted in this pocket in the proper way that minimizes the steric hindrance of the methyl group to the closest residues, Y223 (in the *R* isomer) or I244 (in the *S* isomer), both delineating the proximal region of the acyl channel. Therefore, in principle, these two positions may be potential candidates to introduce the binding preferences of the isomers. However, changes in Y223, which is tightly fixed by the interaction with W228 and W268, as seen in Fig. 6C, might be deleterious for the active site integrity. This, together with the fact that Y223 was found to be less important than I244 (cap domain) according to the evolutionary trace analysis (most important 32%), similar to its interacting tryptophans (W228 and W268 were most important 57% and 22%, respectively), was the basis by which we concentrated our efforts on I244. Its close proximity to the substrates and its prominent position at the long α7- α8 loop suggest a crucial role in binding specificity.

To conclude, our structural analysis of the chiral substrate-bound form of inactivated protein has provided new information explaining the broad substrate promiscuity of EH_3_, which could not be observed previously by examining the crystal structure in free form [39]. Indeed, the results imply that three long channels exist and give access to the catalytic nucleophile, which may then also contribute to the prominent substrate ambiguity of EH_3_ and to its capacity to accept a large variety of esters with different sizes and degrees of conformational dynamics without chiral specificity. In addition, it has also contributed to confirming position 244 as a key position possibly influencing chiral specificity, thus supporting ET prediction.

### 3.5 Position 244 introduces chiral specificity without major influences on substrate ambiguity

To choose which amino acid substitutions of residue I244 to study experimentally, in addition to evolutionary trace analysis, BLAST and structure analyses, we used the Evolutionary Action (EA) method. EA estimates the functional impact of each mutation in a protein and ranks the variants on a scale from 0 (fully neutral) to 100 (fully deleterious) [48], while variants with intermediate scores (e.g., between 40 and 70) have been linked with partial loss or gain of function. In search of gain-of-function effects, we decided to perform two mutations: I244L, which has an EA score of 47 and appears in many homologous sequences (identity up to 66%), and I244F, which is a large amino acid, has an EA score of *ca*. 64, and appears only in distant homologs (E3QWZ9-1, 35% identity as top hit) (Table 3).

**Table 3:** EA scores for mutations in position I244 of EH_3_.

Substitution	Evolutionary Action
I244V	37.83
I244L	46.94
I244M	47.51
I244F	63.58
I244Y	75.39
I244C	75.51
I244T	75.83
I244A	80.26
I244W	81.50
I244N	88.04
I244S	88.93
I244Q	89.28
I244P	89.47
I244H	90.50
I244R	92.53
I244G	93.95
I244K	95.10
I244E	96.97
I244D	97.55

The EH_3I244L_ and EH_3I244F_ variants were created by site-directed mutagenesis, and after expression in the pBXNH3 plasmid and *E. coli* MC1061 cells, the mutants were expressed, purified and characterized using the same protocols as those for the wild-type hydrolase following the hydrolysis of 98 carboxylic ester substrates. Their overall substrate spectra, maximum conversion rates and preferences for chiral esters were evaluated and compared with those of the wild-type protein.

As shown in Fig. 2, Fig. 3, EH_3_ can transform as many as 71 substrates, including chiral and non-chiral substrates, with the highest *k_cat_* of 1730.3 min^−1^; these features were also characteristic of the EH_3I244L_ mutant capable of hydrolyzing the same set of substrates (Fig. 2, Fig. 3) at similar rates (highest *k_cat_* of 1731.3 min^−1^); indeed, the differences in *k_cat_* for the conversion of each ester ranged only from *ca*. 0.7- to 3.2-fold, which suggests no major effects of the mutation on the substrate specificity and conversion rate. The substrate spectrum of EH_3I244F_ was slightly reduced to 53 substrates (Fig. 2, Fig. 3); many large substrates could not be hydrolyzed (such as long alkyl esters or paraben esters), but small substrates such as vinyl acetate and butyrate or propyl propionate and butyrate could be hydrolyzed. Furthermore, when compared to those of the wild type, the *k*_cat_ values of EH_3I244F_ appeared to be lower for most substrates converted, with an average reduction of *ca*. 2.21 (interquartile range from 9.35 to 1.24) and a maximal reduction up to 992-fold (for methyl (*R*)-2-phenylpropanoate). Conversion only increased by *ca*. 2.9-fold for methyl (*S*)-2-phenylpropanoate. These reductions in the substrate repertoire and the conversion rate can be reasonably attributed to the incorporation of a large amino acid residue that does not accommodate as many substrates as wild-type EH_3_ and mutant EH_3I244L_.

Strikingly, the analysis of the *k_cat_* values of separate enantiomers within a series of nine chiral ester couples further revealed significant differences in the preference for chiral esters (Fig. 3). This is exemplified by the apparent significant preference of the EH_3I244F_ mutant for methyl (*S*)-2-phenylpropanoate, (1*R*)-neomethyl acetate, methyl (*S*)-3-hydroxybutyrate, and methyl (*S*)-3-hydroxyvalerate compared to their chiral partners. This contrasts with the wild-type EH_3_ and the EH_3I244L_ mutant, which display no apparent preference for any of the chiral pairs (Fig. 3). As shown in Table 1, the *E*_app_ values of EH_3_ and mutant EH_3I244L_ ranged from 1.02 ± 0.10 to 6.93 ± 0.35 and from 1.04 ± 0.14 to 6.88 ± 0.14, respectively. In contrast, EH_3I244F_ hydrolyzed (1*R*)-neomethyl acetate, methyl (*S*)-3-hydroxybutyrate, and methyl (*S*)-3-hydroxyvalerate, with no appreciable hydrolysis of the other enantiomers detected with our assay conditions, and showed high preferences for methyl (*R*)-lactate (*E*_app_: *ca*. 227 ± 5) and methyl-(*S*)-2-phenylpropanoate (*E*_app_: *ca*. 56300 ± 42) (Table 1); these values are above *E*_app_ > 25, indicative of interest for industrial applications [39].

Encouraged by these promising results, we carried out additional kinetic analyses with separate methyl-2-propanoate enantiomers used for soaking experiments and confirmed the absence of preferences of EH_3_ and EH_3I244L_ at any incubation time (Fig. S3) and the marked preference of EH_3I244F_ for methyl-(*S*)-2-phenylpropanoate. These results were confirmed by measuring the enantiomeric excess (*e.e*.%) with a racemic mixture of methyl-2-propanoate enantiomers by GC [22], with values of 99.99 ± 0.35% for EH_3I244F_, 41.70 ± 0.48% for EH_3_ and 42.5 ± 0.44% for EH_3I244L_.

Collectively, EH_3_ gained stereospecificity properties in the I244F mutant. This increase can be explained by the presence of a bulky residue that impedes the binding or positioning of one of the enantiomers. In the case of the methyl-2-phenylpropanoate substrate, for instance, both isomers could be able, in principle, to properly stack their phenyl moiety against the aromatic F244 side chain (Fig. 6D), but then the (*R*) isomer would probably present high steric hindrance of its methyl group to the Y223 side chain, resulting in a preference for methyl-(*S*)-2-phenylpropanoate binding.

## 4 Conclusions

Although multiple lines of evidence indicate a general trend of enzymes evolving from a generalist ancestor that accepts a broad range of substrates to a specialist enzyme [4], to our knowledge, there is no information on the coevolution of multi-specificity and chiral specificity. Here, combined analyses of specificity through evolutionary trace, structure determination and mutagenesis reveal that substrate ambiguity and chiral specificity in a single hydrolase can be modulated by a single residue. In this way, it is feasible to engineer prominent substrate-promiscuous yet stereospecific hydrolases that are relevant to the field of organic synthesis. We hypothesize that the number of enzymes with such characteristics will increase in the future through screening evolutionarily important single sequence positions, allowing us to swap substrate ambiguity and chiral specificity.

## 5 Accession number

The coordinates and structure factors of EH_3S192A_ complexed with methyl-(*R*/*S*)-2-phenylpropanoate have been deposited in the Protein Data Bank with the accession codes 6SYA and 6SXY.

## CRediT authorship contribution statement

**Isabel Cea-Rama:** Methodology, Formal analysis. **Cristina Coscolín:** Methodology, Formal analysis. **Panagiotis Katsonis:** Methodology, Formal analysis. **Rafael Bargiela:** Formal analysis. **Peter N. Golyshin:** Methodology, Funding acquisition. **Olivier Lichtarge:** Methodology, Funding acquisition. **Manuel Ferrer:** Formal analysis, Resources, Writing - original draft, Funding acquisition. **Julia Sanz-Aparicio:** Formal analysis, Resources, Writing - original draft, Funding acquisition.

## Declaration of Competing Interests

The authors declare that they have no known competing financial interests or personal relationships that could have appeared to influence the work reported in this paper.
