# Supplementary material for: Structure and evolutionary trace-assisted screening of a residue swapping the substrate ambiguity and chiral specificity in an esterase
Source: Comput Struct Biotechnol J. 2021 Apr 18;19:2307–17. doi: 10.1016/j.csbj.2021.04.041 (PMC8105184; doi:10.1016/j.csbj.2021.04.041)
Supplement: Supplementary data 1 [file mmc1.docx]

SUPPLEMENTARY INFORMATION

Structure and evolutionary trace-assisted screening of a residue swapping the substrate ambiguity and chiral specificity in an esterase

Isabel Cea-Rama, Cristina Coscolín, Panagiotis Katsonis, Rafael Bargiela, Peter N. Golyshin, Olivier Lichtarge, Manuel Ferrer, and Julia Sanz-Aparicio

Table of Contents

Figures and Tables

Fig. S1 ……………………………………………………………………………………………………………………… 2

Fig. S2 ……………………………………………………………………………………………………………………… 2

Fig. S3 ……………………………………………………………………………………………………………………… 3

Table S1 …………………………………………………………………………………………………………………… 4

Table S2 …………………………………………………………………………………………………………………… 5

Table S3 …………………………………………………………………………………………………………………… 8

Table S4 …………………………………………………………………………………………………………………… 9


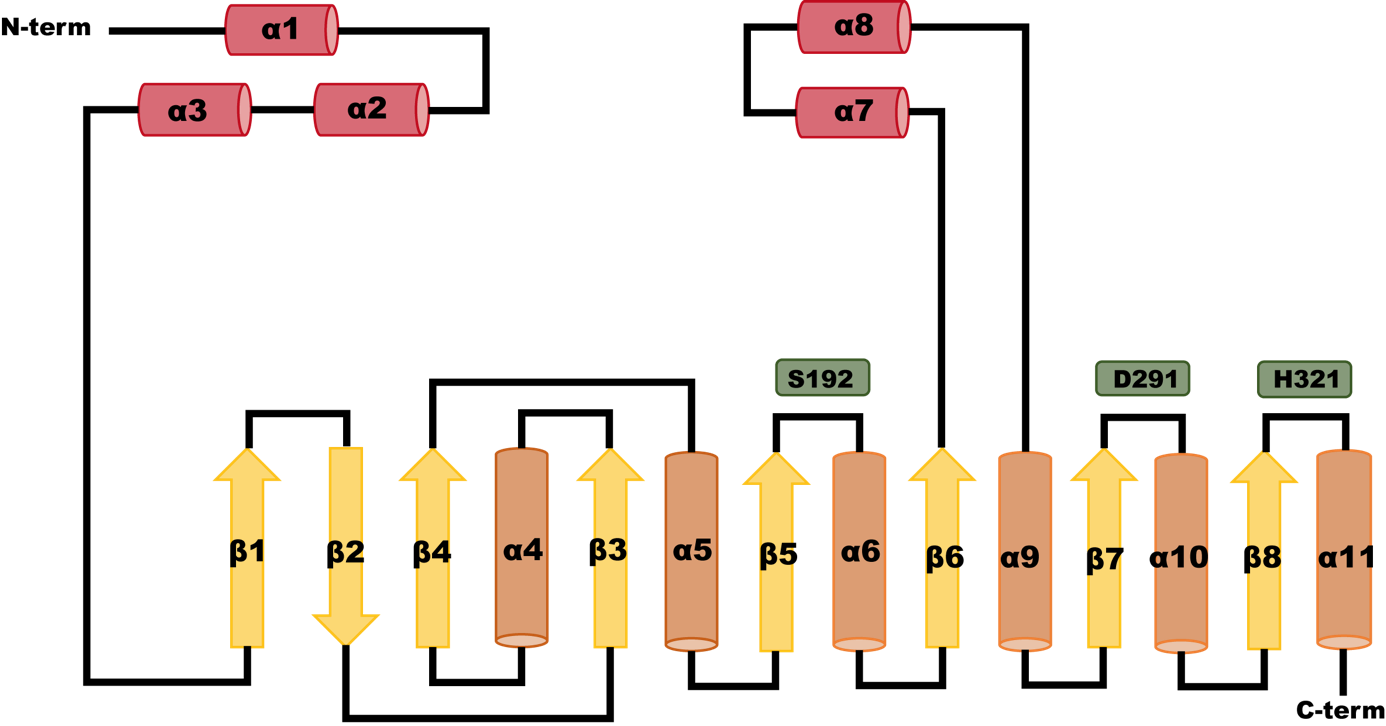


**Fig. S1. Schematic representation of the EH_3_ fold showing the canonical α/β-hydrolase core**. The catalytic triad is depicted as green rectangles.


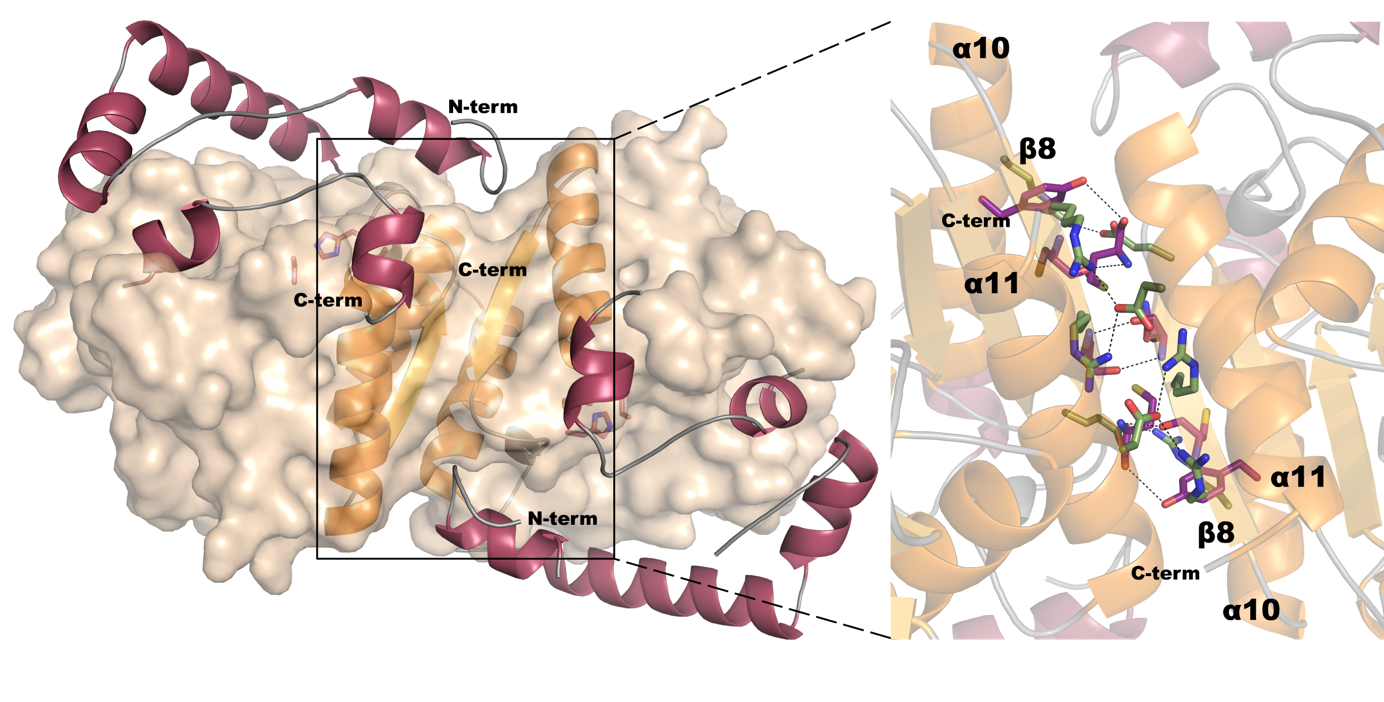


**Fig. S2. The EH_3_ dimer.** The subunits are related by a twofold symmetry axis perpendicular to the picture that situates the two active sites in an opposite face of the dimer, far away from the dimeric interface. The secondary structures involved in the dimeric interface are illustrated as cartoons. A zoom of the dimeric interface showing the hydrogen bond (purple) and salt bridge (green) interactions formed. The dimeric interface occurs by the antiparallel interaction of $\beta$8 strands and additional contacts between $\alpha$10 and $\alpha$11 from both subunits. The interface has an area of 1028.3 Å^2^ (approximately 8% of the total surface). Hydrogen bonds are mainly created between $\beta$8 and $\alpha$10, whereas salt bridges are present in all three secondary structures that build the interface (**Table S3**).

**Fig. S3.** Time course of methyl (*R*/*S*)-2-phenylpropanoate hydrolysis. Kinetic conversion of separate enantiomers of methyl-2-phenylpropanoate (M(*R*)2PP, filled circles; M(*S*)2PP, open circles) by a control test without enzyme (**A**) and the three hydrolytic variants (**B**: EH_3_; **C**: EH_3I244L_; **D**: EH_3I244F_). The kinetics of the hydrolysis of separate enantiomers followed by recordings of the absorbance at 550 nm are shown. A control reaction without enzyme is shown. At each data point, enantiomeric excess (*e.e*.%) was determined by gas chromatography using a racemic mixture of methyl (*R*/*S*)-2-phenylpropanoate. Graphics were created with SigmaPlot version 14.0. Data are not fitted to any model.

**Table S1.** Crystallographic statistics of EH_3S192A_ complexed with methyl-(*R*/*S*)-2-phenylpropanoate.

| (Values in brackets are for the high-resolution shell) | | |
| --- | --- | --- |
| **Crystal data** | EH_3S192A_ – (R) | EH_3S192A_ – (S) |
| Space group | *C2* | *C2* |
| Unit cell parameters |  |  |
| a (Å) | 184.10 | 184.07 |
| b (Å) | 51.17 | 51.71 |
| c (Å) | 70.44 | 70.24 |
| β (°) | 93.96 | 93.76 |
| **Data collection** |  |  |
| Beamline | XALOC(ALBA) (ALBA)MASSIF-1 | XALOC (ALBA) |
| Temperature (K) | 100 | 100 |
| Wavelength (Å) | 0.97924 | 0.97924 |
| Resolution (Å) | 45.91-2.27  (2.34-2.27) | 45.92-2.06  (2.12-2.06) |
| **Data processing** |  |  |
| Total reflections | 162231 (15254) ((((17149) (17149) (17149) | 218980 (17088) |
| Unique reflections | 30378 (2810) | 40816 (3159) |
| Multiplicity | 5.3 (5.4) | 5.4 (5.4) |
| Completeness (%) | 99.4 (99.7) | 99.4 (99.8) |
| Mean *I*/σ (*I*) | 6.6 (3.1) | 8.9 (4.6) |
| *R_merge_^†^* (%) | 17.2 (65.6) | 13.7 (57.3) |
| *R_pim_^††^* (%) | 8.1 (30.2) | 6.6 (28.6) |
| Molecules per ASU | 2 | 2 |
| **Refinement** |  |  |
| R_work_/R_free_*^†††^* (%) | 21.0/24.0 | 19.2/22.8 |
| **N° of atoms/average B** (Å^2^) | 5478/30.26 | 5534/28.14 |
| Macromolecule | 5158/29.97 | 5145/27.63 |
| Ligands | 72/47.74 | 63/51.03 |
| Solvent | 248/31.36 | 326/31.70 |
| **Ramachandran plot** (%) |  |  |
| Favored (%) | 95.1 | 95.5 |
| Outliers (%) | 0.1 | 0.1 |
| **RMS deviations** |  |  |
| Bonds (Å) | 0.004 | 0.006 |
| Angles (°) | 1.413 | 1.483 |
| **PDB accession codes** | 6SYA | 6SXY |

^†^R_merge_ = ∑_hkl_ ∑_i_ | I_i_(hkl) – [I(hkl)]| / ∑_hkl_ ∑_i_  I_i_(hkl), where I_i_(hkl) is the ith measurement of reflection hkl and [I(hkl)] is the weighted mean of all measurements.

^††^R_pim_ = ∑_hkl_ [1/(N - 1)] 1/2 ∑_i_ | I_i_(hkl) – [I(hkl)]| / ∑_hkl_ ∑_i_  I_i_(hkl), where N is the redundancy for the hkl reflection.

^†††^R_work_ / R_free_ = ∑_hkl_ | Fo – Fc | / ∑_hkl_ | Fo |, where Fc is the calculated and Fo is the observed structure factor amplitude of reflection hkl for the working / free (5%) set.

**Table S2.** *k_cat_* values. List of carboxyl ester substrates found to be converted by EH_3_ and its mutants. Shown are the *k_cat_* values for a number of non-chiral and chiral esters. The following information is provided: name of the ester, *LogP* value of the ester calculated using ACD/ChemSketch 2015.2.5 software, molecular volume of the ester calculated as described in <http://www.molinspiration.com/cgi-bin/properties>, and average *k_cat_* in min^-1^. The assays were performed at 30°C and pH 8.0. The results correspond to the average values of three assays. The standard deviation was less than 1% in all cases.

|  |  |  |  |  | ***k_cat_* (min^-1^)** | | |
| --- | --- | --- | --- | --- | --- | --- | --- |
| **Non-chiral esters** | **Log P** | **Volume [Å^3^]** | **Mw [g/mol]** | **Smiles code** | **EH_3_** | **EH_3I243L_** | **EH_3I243F_** |
| 1-Naphthyl acetate | 2.65 | 172.56 | 186,21 | CC(=O)OC1=CC=CC2=CC=CC=C21 | 1051.64 | 1052.82 | 1028.07 |
| 1-Naphthyl butyrate | 3.88 | 206.17 | 214,26 | CCCC(=O)OC1=CC=CC2=CC=CC=C21 | 148.66 | 149.78 | 109.45 |
| Glyceryl triacetate | 0.52 | 196.65 | 218,20 | CC(=O)OCC(COC(=O)C)OC(=O)C | 1475.89 | 1477.29 | 1305.38 |
| Glyceryl tripropionate | 1.6 | 247.06 | 260,28 | CCC(=O)OCC(COC(=O)CC)OC(=O)CC | 1730.26 | 1731.29 | 1107.86 |
| Glyceryl tributyrate | 3.27 | 297.46 | 302,36 | CCCC(=O)OCC(COC(=O)CCC)OC(=O)CCC | 1504.05 | 1505.07 | 1221.30 |
| Hexyl acetate | 2.83 | 157.73 | 144,21 | CCCCCCOC(=O)C | 114.25 | 115.24 | 3206.94 |
| Octyl acetate | 3.84 | 191.34 | 172,26 | CCCCCCCCOC(=O)C | 171.00 | 172.10 | 145.35 |
| Ethyl propionate | 1.12 | 107.33 | 102,13 | CCC(=O)OCC | 22.94 | 24.53 | 18.35 |
| Ethyl butyrate | 1.68 | 124.13 | 116,16 | CCCC(=O)OCC | 20.38 | 21.42 | 1.31 |
| Ethyl hexanoate | 2.69 | 157.73 | 144,21 | CCCCCC(=O)OCC | 57.81 | 58.67 | 266.70 |
| Ethyl octanoate | 3.70 | 191.34 | 172,26 | CCCCCCCC(=O)OCC | 90.71 | 91.52 | 27.88 |
| Ethyl decanoate | 4.71 | 224.94 | 200,32 | CCCCCCCCCC(=O)OCC | 9.06 | 9.87 | 0.33 |
| Ethyl dodecanoate | 5.72 | 258.55 | 228,37 | CCCCCCCCCCCC(=O)OCC | 0.60 | 1.59 | 0.00 |
| Ethyl benzoate | 2.48 | 145.37 | 150,17 | CCOC(=O)C1=CC=CC=C1 | 151.38 | 152.37 | 0.90 |
| Benzoic acid, 4-formyl-, phenylmethyl ester | 3.49 | 219.21 | 240,25 | C1=CC=C(C=C1)COC(=O)C2=CC=C(C=C2)C=O | 263.22 | 263.99 | 171.13 |
| Propylparaben | 2.51 | 170.19 | 180,20 | CCCOC(=O)C1=CC=C(C=C1)O | 62.33 | 0.13 | 0.00 |
| Butylparaben | 3.07 | 187.00 | 194,23 | CCCCOC(=O)C1=CC=C(C=C1)O | 98.56 | 0.14 | 0.00 |
| Phthalic acid diethyl ester | 2.31 | 206.71 | 222,24 | CCOC(=O)C1=CC=CC=C1C(=O)OCC | 243.90 | 244.70 | 0.00 |
| Benzyl (*R*)-(+)-2-hydroxy-3-phenylpropionate | 2.93 | 241.87 | 256,30 | C1=CC=C(C=C1)CC(C(=O)OCC2=CC=CC=C2)O | 647.78 | 648.77 | 0.00 |
| Benzylparaben | 3.22 | 208.24 | 228,24 | C1=CC=C(C=C1)COC(=O)C2=CC=C(C=C2)O | 68.82 | 0.33 | 0.00 |
| Methyl benzoate | 2.11 | 128.57 | 136,15 | COC(=O)C1=CC=CC=C1 | 71.24 | 72.46 | 0.00 |
| Methyl hexanoate | 2.31 | 140.93 | 130,18 | CCCCCC(=O)OC | 65.35 | 0.00 | 0.00 |
| Methyl octanoate | 3.33 | 174.54 | 158,24 | CCCCCCCC(=O)OC | 121.19 | 122.15 | 19.43 |
| Propyl propionate | 1.62 | 124.13 | 116,16 | CCCOC(=O)CC | 7.70 | 8.57 | 0.00 |
| Propyl butyrate | 2.18 | 140.93 | 130,18 | CCCC(=O)OCCC | 8.30 | 9.23 | 0.00 |
| Propyl hexanoate | 3.19 | 174.54 | 158,24 | CCCCCC(=O)OCCC | 161.79 | 162.81 | 282.25 |
| Phenylethyl cinnamate | 4.33 | 244.44 | 252,31 | C1=CC=C(C=C1)CCOC(=O)C=CC2=CC=CC=C2 | 32.90 | 33.83 | 0.00 |
| Isobutyl cinnamate | 3.65 | 206.18 | 204,26 | CC(C)COC(=O)C=CC1=CC=CC=C1 | 82.56 | 83.59 | 7.56 |
| Methyl cinnamate | 2.53 | 155.99 | 162,19 | COC(=O)C=CC1=CC=CC=C1 | 161.64 | 162.71 | 3.62 |
| Methyl ferulate | 1.86 | 189.55 | 208,21 | COC1=C(C=CC(=C1)C=CC(=O)OC)O | 25.20 | 26.37 | 0.00 |
| Vinyl acetate | 0.69 | 84.89 | 86,09 | CC(=O)OC=C | 13.13 | 14.01 | 0.30 |
| Vinyl propionate | 1.05 | 101.70 | 100,12 | CCC(=O)OC=C | 2.87 | 3.53 | 0.00 |
| Vinyl butyrate | 1.61 | 118.50 | 114,14 | CCCC(=O)OC=C | 73.80 | 74.69 | 0.00 |
| Vinyl benzoate | 2.41 | 139.74 | 148,16 | C=COC(=O)C1=CC=CC=C1 | 346.23 | 347.10 | 278.58 |
| Vinyl crotonate | 1.37 | 112.31 | 112,13 | CC=CC(=O)OC=C | 268.95 | 269.85 | 3713.16 |
| Vinyl acrylate | 1.12 | 96.06 | 98,10 | C=CC(=O)OC=C | 1.96 | 2.83 | 0.00 |
| Geranyl acetate | 3.91 | 212.09 | 196,29 | CC(=CCCC(=CCOC(=O)C)C)C | 212.20 | 213.05 | 522.09 |
| 3-Methyl-3-buten-1-yl acetate | 1.85 | 135.06 | 128,17 | CC(=C)CCOC(=O)C | 521.00 | 521.84 | 503.47 |
| Ethyl 2-ethylacetoacetate | 1.34 | 159.70 | 158,19 | CCC(C(=O)C)C(=O)OCC | 225.18 | 226.25 | 183.27 |
| Ethyl 2-methylacetoacetate | 0.84 | 142.90 | 144,17 | CCOC(=O)C(C)C(=O)C | 255.82 | 256.67 | 67.34 |
| Ethyl 3-oxohexanoate | 1.12 | 159.92 | 158,19 | CCCC(=O)CC(=O)OCC | 498.97 | 500.01 | 401.42 |
| Ethyl acetoacetate | 0.06 | 126.31 | 130,14 | CCOC(=O)CC(=O)C | 526.13 | 526.94 | 121.47 |
| Ethyl propionylacetate | 0.56 | 143.11 | 144,17 | CCC(=O)CC(=O)OCC | 523.42 | 524.23 | 322.10 |
| ϒ-Valerolactone | -0.46 | 96.75 | 100,12 | CC1CCC(=O)O1 | 54.94 | 55.65 | 2.20 |
| Methyl glycolate | -0.46 | 81.98 | 90,08 | COC(=O)CO | 288.88 | 290.02 | 31.96 |
| Ethyl 2-chlorobenzoate | 2.78 | 158.91 | 184,62 | CCOC(=O)C1=CC=CC=C1Cl | 464.55 | 465.38 | 130.40 |
| Cyclohexyl butyrate | 3.21 | 180.76 | 170,25 | CCCC(=O)OC1CCCCC1 | 296.57 | 297.48 | 690.15 |
| n-Pentyl benzoate | 4.05 | 195.78 | 192,26 | CCCCCOC(=O)C1=CC=CC=C1 | 66.26 | 67.07 | 16.93 |
| Propyl acetate | 1.26 | 107.33 | 102,13 | CCCOC(=O)C | 1514.96 | 1515.80 | 71.07 |
| Butyl acetate | 1.82 | 124.13 | 116,16 | CCCCOC(=O)C | 1557.83 | 1558.72 | 205.86 |
| Phenyl acetate | 1.49 | 128.57 | 136,15 | CC(=O)OC1=CC=CC=C1 | 1542.13 | 1543.00 | 1233.32 |
| Phenyl propionate | 2.16 | 145.37 | 150,17 | CCC(=O)OC1=CC=CC=C1 | 1502.59 | 1503.16 | 1204.15 |
| Glucose pentaacetate | 0.44 | 338.38 | 390,34 | CC(=O)OCC(C(C(C(C=O)OC(=O)C)OC(=O)C)OC(=O)C)OC(=O)C | 1550.28 | 1550.96 | 895.09 |
| **Chiral esters** |  |  |  |  |  |  |  |
| (1*R*)-Menthyl acetate | 4.04 | 213.72 | 198.30 | CC1CCC(C(C1)OC(=O)C)C(C)C | 84.52 | 85.19 | 39.90 |
| (1*S*)-Menthyl acetate | 4.04 | 213.72 | 198.30 | CC1CCC(C(C1)OC(=O)C)C(C)C | 57.81 | 58.49 | 6.55 |
| Methyl (*R*)-mandelate | 0.99 | 153.42 | 166.17 | COC(=O)C(C1=CC=CC=C1)O | 214.32 | 215.02 | 8.22 |
| Methyl (*S*)-mandelate | 0.99 | 153.42 | 166.17 | COC(=O)C(C1=CC=CC=C1)O | 141.57 | 142.29 | 23.42 |
| Ethyl (*R*)-4-chloro-3-hydroxybutyrate | 0.47 | 145.95 | 166.60 | CCOC(=O)CC(CCl)O | 185.94 | 186.77 | 4.85 |
| Ethyl (*S*)-4-chloro-3-hydroxybutyrate | 0.47 | 145.95 | 166.60 | CCOC(=O)CC(CCl)O | 223.52 | 224.13 | 38.35 |
| Ethyl *D*-Lactate | 0.28 | 115.37 | 118.13 | CCOC(=O)C(C)O | 318.16 | 318.94 | 21.75 |
| Ethyl *L*-lactate | 0.28 | 115.37 | 118.13 | CCOC(=O)C(C)O | 324.49 | 325.27 | 30.50 |
| Methyl (*S*)-3-hydroxybutyrate | -0.13 | 115.37 | 118.13 | CC(CC(=O)OC)O | 6.34 | 7.01 | 0.19 |
| Methyl (*R*)-3-hydroxybutyrate | -0.13 | 115.37 | 118.13 | CC(CC(=O)OC)O | 38.64 | 39.41 | 0.00 |
| (1*R*)-Neomenthyl acetate | 4.04 | 213.72 | 198.30 | CC1CCC(C(C1)OC(=O)C)C(C)C | 169.19 | 169.97 | 125.70 |
| (1*S*)-Neomenthyl acetate | 4.04 | 213.72 | 198.30 | CC1CCC(C(C1)OC(=O)C)C(C)C | 37.43 | 38.11 | 0.00 |
| Methyl (*R*)-3-hydroxyvalerate | 0.37 | 132.17 | 132.16 | CCC(CC(=O)OC)O | 129.34 | 130.23 | 0.00 |
| Methyl (*S*)-3-hydroxyvalerate | 0.37 | 132.17 | 132.16 | CCC(CC(=O)OC)O | 59.47 | 59.99 | 23.21 |
| Methyl *D*-Lactate | -0.09 | 98.57 | 104.10 | CC(C(=O)OC)O | 1431.65 | 1432.20 | 1483.26 |
| Methyl *L*-Lactate | -0.09 | 98.57 | 104.10 | CC(C(=O)OC)O | 1212.36 | 1213.04 | 81.52 |
| Methyl (*R*)-2-phenylpropanoate | 2.14 | 161.96 | 164.2 | CC(C1=CC=CC=C1)C(=O)OC | 111.54 | 112.25 | 0.11 |
| Methyl (*S*)-2-phenylpropanoate | 2.14 | 161.96 | 164.2 | CC(C1=CC=CC=C1)C(=O)OC | 212.51 | 213.20 | 621.60 |

**Table S3.** Atomic interactions at the EH_3_ dimer interface.

| Hydrogen bonds | | | Salt bridges | | |
| --- | --- | --- | --- | --- | --- |
| Chain B | Dist. (Å) | Chain A | Chain B | Dist. (Å) | Chain A |
| M317 [N] | 3.0 | C313 [O] | R302 [NH_2_] | 2.6 | E289 [OE_2_] |
| Q315 [N] | 2.9 | Q315 [O] | R312 [NH_1_] | 2.9 | D336 [OD_2_] |
| Y301 [OH] | 3.6 | M317 [O] | R314 [NH_2_] | 2.9 | D336 [OD_2_] |

**Table S4.** ET scores for all EH_3_ residues.

| Residue | AA type | AA variability | ET (% rank) |
| --- | --- | --- | --- |
| 1 | M | .MRKDVNSAEQITPLHG | 77 |
| 2 | P | .PMLHIVRTEDANWKQSGCFY | 88 |
| 3 | D | .DQGAIFTSRKEMHLPVYN | 89 |
| 4 | T | .TSNDARKVQGFLEMPHIC | 77 |
| 5 | T | MT.SPAWHLEYGFNQVDKRI | 76 |
| 6 | S | PSMTKQNEDR.GVLAFYHI | 100 |
| 7 | L | NLSYDCMIF.WPKAHRGQETV | 85 |
| 8 | N | KNDRSTVQE.IGMYAPLFCH | 76 |
| 9 | I | LIMPFV.DTNHSKAEWQRGYC | 84 |
| 10 | A | SANMDKGRYFE.ILVTPWQH | 82 |
| 11 | D | ADEQNTHGRMIS.PVKLFYW | 71 |
| 12 | D | DETKVAPSGRNYMILH.FQ | 70 |
| 13 | V | KVPDRAQSGHNFLWIETY.M | 41 |
| 14 | R | RSYAGLKQPNDEMIVH.TFC | 66 |
| 15 | M | IMVLTSAFCRYE.PKDWG | 52 |
| 16 | D | DHNQVSLPARI.KTEFGM | 25 |
| 17 | P | PMRAISQE.KNFLDTHGV | 39 |
| 18 | R | RGKNADEFYIQTVS.PHL | 56 |
| 19 | L | ILNMEFYVWGSR.HTACK | 32 |
| 20 | K | KRLVIATFDYQSEG.WM | 65 |
| 21 | A | AEGKPD.HV | 66 |
| 22 | M | VMLIGATKSNQDRPWEY.FH | 78 |
| 23 | L | FLMTIAEGQVPRWKY.SN | 67 |
| 24 | A | GASEDTKRLVFMHINW.QCPY | 32 |
| 25 | A | EASGKQPRTDVFLN.IMHW | 81 |
| 26 | F | LFMIYAQVSENHPRGT.DK | 81 |
| 27 | P | GPDESQRLKIAFMVY.WTCNH | 69 |
| 28 | M | IMVLSGQWFC.PARHNDTKEY | 74 |
| 29 | M | PMAGT.DEQKSRLYNIVF | 77 |
| 30 | E | VEAGLDM.RKNITHYFQS | 84 |
| 31 | Q | TQAGVSPFYIERKL.MHND | 80 |
| 32 | Q | QKGRVSNDL.PEIFTYMAHC | 86 |
| 33 | T | KTNDASVPGLEQ.IRMFHY | 51 |
| 34 | F | AFVPGYSEITKL.MNWDHRQC | 72 |
| 35 | Q | AQEPDLIVSKYCTN.GRHWFM | 71 |
| 36 | T | STNDAGRQH.PKLVIFYEMCW | 82 |
| 37 | R | RATVHEMI.KDSLGPQYNF | 74 |
| 38 | E | DENALSITRKGPVHQ.MCFY | 74 |
| 39 | E | EKAVI. | 84 |
| 40 | Q | IQLMATSRDVWNKF.HEGP | 63 |
| 41 | V | MVLPSEQDNFHIYAR.TWGK | 56 |
| 42 | A | EADLVSMHFQITKR.PNGW | 74 |
| 43 | N | RNAVDETLPCKSGH.QMIF | 79 |
| 44 | A | EAVDQGSCLYKRWMFTN.IPH | 66 |
| 45 | N | NSIRCLKMTAV.QPHD | 19 |
| 46 | T | STRILHQWAMNEDVK.PGF | 84 |
| 47 | P | EPAQKTSNG.RILDMFHVWY | 72 |
| 48 | E | AEKFYSTQGIN.MLDWVRHP | 76 |
| 49 | A | ASGELCMVI.RTHQNKFDYPW | 78 |
| 50 | T | VTLIHASMGKRE.DNQP | 95 |
| 51 | A | QAMEILHNKWDY.TFCPRG | 81 |
| 52 | A | EARVTKSPYMQN.CGILFDH | 90 |
| 53 | R | RMEADNHSL.GITPKQVFY | 82 |
| 54 | E | AENDQKTSYV.LPRFGM | 87 |
| 55 | Q | IQEGMATNKSDPCR.VLFHYW | 81 |
| 56 | L | MLIVTSFWHKRDAYN.QGEPC | 71 |
| 57 | K | QKTRGSEVNMAIFLD.PHYW | 75 |
| 58 | M | LMAVKEGSTNRDHIQ.FCYPW | 86 |
| 59 | M | IMFLVAGPEYSR.TNQHDKW | 71 |
| 60 | M | NMFLIHYAESPGT.DRVKQW | 72 |
| 61 | D | NDEGHSAKTRY.PMFVQILC | 81 |
| 62 | M | AMLVGTSIFDKENPQR.Y | 84 |
| 63 | M | PMACLSVYRNQEHITWGD.KF | 76 |
| 64 | D | HDAGV.PKLYFETSRIN | 49 |
| 65 | S | YSNDFARLGMP.KITVQHE | 85 |
| 66 | E | EQIGAKSDTV.LYPHRNMF | 80 |
| 67 | E | EQDKSVALMPGINFT.RHY | 71 |
| 68 | F | VFILPAEHS.DTKGYMNRWQ | 75 |
| 69 | A | IATVHSPRDGQE.NCKFLMY | 75 |
| 70 | P | TPVSDIR.LMYFACGHWEKQN | 57 |
| 71 | S | YSKNAPHVQDE.L | 93 |
| 72 | D | EDAKQGPLITNF. | 95 |
| 73 | N | GNQ.ERL | 27 |
| 74 | L | LS.PGQDAN | 47 |
| 75 | D | VDSKIRTLAEHCYPF.MQWG | 72 |
| 76 | I | ITASDEVKHLNCRFMQ.PWG | 76 |
| 77 | S | STEAVCHYRQDNIK.LFWPG | 68 |
| 78 | T | ETDSKRIVLYFHQA.GMCW | 79 |
| 79 | R | KRHYEQMTVNSALFPIDW.CG | 85 |
| 80 | E | ETSVAQMYLIWF.RNKPC | 50 |
| 81 | F | FVITKE.LRCAPNDSQHG | 82 |
| 82 | T | ITVKQDAFY.ERLGSH | 78 |
| 83 | S | SPTKENRGDAM.QVLI | 69 |
| 84 | S | ESQDAGTNFVR.LPKHIY | 42 |
| 85 | P | PVTASRLFQWNEDKHYG.M | 64 |
| 86 | D | DNGS.RQHLAPKETIV | 51 |
| 87 | G | GEAN.DHSVKRLIYQCTM | 26 |
| 88 | N | NHRTSYA.GQDVECKPLF | 67 |
| 89 | A | TAISQEVDLKP.YFGRMNH | 81 |
| 90 | I | VIAMR.LPTNQF | 37 |
| 91 | K | KNHDQTPRAVM.ELGISY | 66 |
| 92 | I | ILMATV.CGFYS | 45 |
| 93 | Q | QSNFYHKTVWRALCED. | 25 |
| 94 | F | YFIVLARWEHTCSMN.G | 64 |
| 95 | I | IMYSAHFVTLPC. | 24 |
| 96 | R | RSEKQTYFAMGLNIVHDW. | 57 |
| 97 | P | PR.KTSGVANDQI | 10 |
| 98 | K | DKRNG.TSQALE | 75 |
| 99 | G | TGSNAVHRE.LQM | 96 |
| 100 | K | DKTSQGENRP.AHLVCI | 90 |
| 101 | Q | EQDAGTSRKH.NPLVM | 90 |
| 102 | K | TKHIVMEPADLSQ.NGR | 100 |
| 103 | V | LVIRSGTENAKQMDFHP.WY | 51 |
| 104 | P | PALKSGCRIVT.QD | 12 |
| 105 | C | CAGVITLRM.SP | 40 |
| 106 | V | VILFAWMYTGH | 45 |
| 107 | Y | YFLVIMCAS.G | 33 |
| 108 | Y | YHMWNRDFSGQIVKAL.T | 26 |
| 109 | I | IFLTV.MHASCY | 46 |
| 110 | H | HTPY | 2 |
| 111 | G | GAPSM | 3 |
| 112 | G | GSA | 2 |
| 113 | G | GAR.VCFS | 3 |
| 114 | M | MIVYFWTLHQ.G | 15 |
| 115 | M | EMQAVCTPF.ISLG | 37 |
| 116 | I | MISNTFV.LGEAQRDY | 61 |
| 117 | M | SMLYAGI.TNVHERCFQ | 10 |
| 118 | S | SDTQRNK.EPFILVGHMAWY | 53 |
| 119 | A | CATR.LVPGINHQEYFDSKWM | 37 |
| 120 | F | FYLQAEMI.SDKPVRNTHG | 64 |
| 121 | Y | DYNFGTS.RVAQILHMWCE | 50 |
| 122 | G | GARVLPK.HDFYCSENMQITW | 28 |
| 123 | N | INLTGAV.F | 70 |
| 124 | Y | YHS.NV | 21 |
| 125 | R | KRQTAVGS.DFNLEMIHP | 44 |
| 126 | A | ATSHR.YNLFKPIGDEVQCW | 70 |
| 127 | W | WYIL.NTFMDVSAPGRQEK | 58 |
| 128 | G | GARCL.IVTMHNFYSP | 40 |
| 129 | K | RKEDNSTCHQLIAVGFM.WP | 42 |
| 130 | M | CMILTASNHDEGPQRKFVY.W | 75 |
| 131 | I | IMLQWVYRFSTNHDA. | 29 |
| 132 | A | ATSIVCML.P | 17 |
| 133 | N | LNASHRTIQVEKDG.YFWCM | 81 |
| 134 | N | QNTKMASRLEVDGP.HIWFY | 76 |
| 135 | G | GNKADPESRQH.L | 50 |
| 136 | V | VLMTGIFAHC.QSYWR | 53 |
| 137 | A | ACVLMSITPQHYR.EKDG | 42 |
| 138 | V | VALCSTIFMQG. | 22 |
| 139 | A | AVMILFYRCS | 31 |
| 140 | M | MLGFSAYTVDNHC | 20 |
| 141 | V | VLIYAPMF.T | 11 |
| 142 | D | DENKGPRAQ.THVS | 25 |
| 143 | F | FHY.W | 3 |
| 144 | R | RSGNHTCDEQ | 5 |
| 145 | N | NSLKTVWRMHCPQ | 10 |
| 146 | C | SCAVR. | 41 |
| 147 | L | RLVGAWYF. | 66 |
| 148 | S | VSTR.L | 85 |
| 149 | P | P. | 21 |
| 150 | S | S. | 21 |
| 151 | S | SA.T | 44 |
| 152 | A | SALVGDTCP | 10 |
| 153 | P | GPENRKTALSI | 6 |
| 154 | E | EGLTKAHQDNS | 8 |
| 155 | V | VNGEQLIKYSWHFRCATD | 43 |
| 156 | A | APNFYHR.G | 51 |
| 157 | P | PAQKTSRHGVLIEC | 25 |
| 158 | F | FYHADQGRVWCP | 18 |
| 159 | P | PTLG | 1 |
| 160 | A | ATIVNLGHRYQKCWE | 28 |
| 161 | G | GAPQDLW | 16 |
| 162 | L | LSFQGVHIARPNWYTM | 23 |
| 163 | N | NDTHLKQEGRSVFYI | 55 |
| 164 | D | DQE | 4 |
| 165 | C | CVGSAFNIDYTL | 21 |
| 166 | V | VIATCKGFYWMQHELSDR | 34 |
| 167 | S | SADTERNYLMFGKQV | 45 |
| 168 | G | GACVTSIDLM | 26 |
| 169 | L | ILVYFATWMGS | 26 |
| 170 | K | KRQLHAENVCTISDGM | 78 |
| 171 | W | WYHELASKFID | 9 |
| 172 | V | LVATIMFSCQG | 44 |
| 173 | S | HSVATYNIDWLMFRQKGCE. | 68 |
| 174 | E | NESAQDKLTRGVH.I | 81 |
| 175 | N | NQHRGEDKSTLYAF.IV | 60 |
| 176 | A | CASLKRGYTPMIEH.QNV | 28 |
| 177 | D | SDEQAGKNLTHR.VPFMY | 86 |
| 178 | E | QEASTDKGR.IYNLMPWVFH | 84 |
| 179 | L | LFYEI.GHVMKRW | 27 |
| 180 | S | NSGARQK.MCHWDPEY | 38 |
| 181 | I | IVTLCFG.AWPRSD | 44 |
| 182 | D | NDQ.SREAVTPKHIG | 18 |
| 183 | K | AKSGP.TRVDILQFNECH | 66 |
| 184 | N | GNQSDAIRTHKEMYCLV | 100 |
| 185 | K | KHRQTNSAGELPDMV | 40 |
| 186 | I | IMLVFTAC | 35 |
| 187 | I | IMLVTAYGSFC | 32 |
| 188 | I | IVLMTASFG | 24 |
| 189 | A | ASHCQTEMFGVLWIYPN | 40 |
| 190 | G | GS | 1 |
| 191 | E | EDRNVIHGCAKQPLSTMF | 21 |
| 192 | S | SGDTC | 3 |
| 193 | G | GATSVM | 6 |
| 194 | G | GR | 0 |
| 195 | G | GAS | 9 |
| 196 | N | NRTGHLACSQMK | 9 |
| 197 | L | LIFHM | 7 |
| 198 | T | TASGVCI | 20 |
| 199 | L | ILCFAVTN.QMS | 16 |
| 200 | A | ATGSVCMIYL | 22 |
| 201 | T | TAVLSCIMFN | 36 |
| 202 | G | GTAVMCSNLPI | 41 |
| 203 | L | LMHISQAVNTRY.F | 26 |
| 204 | K | RKQALMYIHSEDNGVTC.FW | 55 |
| 205 | L | LASCVNTDQKRFYMI. | 20 |
| 206 | K | KNILSRVFHYQAEMTG. | 18 |
| 207 | Q | QNARSK.GDTELIHP | 62 |
| 208 | D | DE.NILFMGTH | 38 |
| 209 | G | NGSR.EPVTADHLFIK | 34 |
| 210 | N | ANDSEHTWYK.QVG | 59 |
| 211 | I | LIQSAGKPHMVF.EDYNTRW | 70 |
| 212 | D | NDGETAPCKSHIQVYRFLMW | 49 |
| 213 | L | ILKTEQGMDAPWRNSVHFC.Y | 76 |
| 214 | V | IVPQLFRAMDHN | 34 |
| 215 | K | TKRQSVADCLI.PFHYNWME | 59 |
| 216 | G | GKAQRF.LYMHCSTNV | 33 |
| 217 | L | LIVAFQMNDG | 8 |
| 218 | Y | YFTVMIGALNWHSCQ | 42 |
| 219 | A | PASGLVECMTFI | 7 |
| 220 | L | LMVHFQCSYIARDGNEW | 51 |
| 221 | C | CDSIVAYNFTGLMW | 12 |
| 222 | P | PAG | 1 |
| 223 | Y | YFMSNCWAEVQTIPLGD | 32 |
| 224 | I | ILTVFCSADYP | 23 |
| 225 | A | ALSNC.GDVQEHRTYM | 15 |
| 226 | G | GNAP.YHDELMTVIFSWQRC | 36 |
| 227 | K | IKASQERLGV.TPYNHF | 45 |
| 228 | W | WYGT.SENPQVMLCHFKRDAI | 55 |
| 229 | P | PD.NAGH | 29 |
| 230 | Q | LQSRD.WG | 55 |
| 231 | D | PDEVS. | 73 |
| 232 | R | ERDA.KQT | 74 |
| 233 | F | NFYSALT.DIHVQ | 51 |
| 234 | P | PQTAD.SKMIEVFLNGCRHY | 65 |
| 235 | S | SRF.PDQTNAIMELHGY | 9 |
| 236 | S | SLMRQH.PYIVWFKAENDT | 33 |
| 237 | S | VSTIRYFLKEPNGH.AQWDMC | 83 |
| 238 | E | EDALKPHQ.RGMSTINYVF | 53 |
| 239 | N | NCHLQFRDISAEVYTMP.KWG | 32 |
| 240 | N | ENDHKQGSALPIVRTFM.CY | 58 |
| 241 | G | GEKADSYNPQHRFTVMLI | 67 |
| 242 | I | ILY.PKFACGRNSMVTDHQWE | 37 |
| 243 | M | LMFDYIWTVASPGH.QNR | 48 |
| 244 | I | LIVTFWHGASMPN | 12 |
| 245 | E | SEDTPNQYKCHG.VAR | 46 |
| 246 | L | LVICRMTAYQDGEKHSPNF | 69 |
| 247 | H | HAGSQEKDTPINVRLMYF | 84 |
| 248 | N | NHSQVTYFGMAIDLWKER | 70 |
| 249 | N | SNCALVMTQGYIDRE | 32 |
| 250 | Q | NQHLSADGETVKRIP.FYM | 78 |
| 251 | G | GTILAYFVKQSWNRMEH.DCP | 53 |
| 252 | A | PATSGVQYLWFNREICHMKD | 41 |
| 253 | L | MLVRSIKHQGFEWNATDYCP | 73 |
| 254 | A | AGILPVYFSDCTMWHQNEKR | 59 |
| 255 | Y | YLPAVFMITNHS | 7 |
| 256 | G | GDTACS.NV | 36 |
| 257 | I | IMHPGFYLAQTRCKVESD | 31 |
| 258 | E | KEADQSTN.GPRVIHYL | 56 |
| 259 | Q | EQAGTSDIVKCRHPN. | 82 |
| 260 | L | LFYKTREDAGP.SQNVMHCI | 85 |
| 261 | E | ELFHNDS.QPAKGVTWRY | 70 |
| 262 | A | NAMKRSLTIGV.PHYWDEQF | 79 |
| 263 | E | KEGRQNDVISTHYLAPM. | 58 |
| 264 | N | NDQTSPHGIRMKA.LEY | 57 |
| 265 | P | PRASIE.QMYVDCGLTKWFH | 38 |
| 266 | L | LMVFTIYHQGCEW.RKSANDP | 32 |
| 267 | A | ACFVYTLQINHMS | 20 |
| 268 | W | WFSVA.NHCTRMYQLDIE | 21 |
| 269 | P | PADI.VLC | 6 |
| 270 | S | GSDYMFHLPANIVERWT | 29 |
| 271 | F | FWAMHYSLT.RIKNQEDV | 37 |
| 272 | A | CAKD.SMVTRLQGHEIYF | 17 |
| 273 | S | STGAMNDPQEKV.HRCY | 74 |
| 274 | A | VAENDKTLRISH.PQG | 57 |
| 275 | E | DEASQVK.PNHRGT | 74 |
| 276 | D | DEM.TSNAQVLYRI | 47 |
| 277 | M | VMLCKAYFRHISPT | 28 |
| 278 | Q | TQKSACREVDNGHYP | 79 |
| 279 | G | GDHNKSRVAQYEM | 21 |
| 280 | L | LFMVQA | 4 |
| 281 | P | PVAG | 3 |
| 282 | P | RPEYKDHSTAVQG | 13 |
| 283 | T | THFSALMVIG | 19 |
| 284 | V | YVFMHITALWRSC | 30 |
| 285 | I | IVLMTSFNA | 30 |
| 286 | N | VNSMAREIDLYGFCTQH | 48 |
| 287 | V | VLMSICTANG | 16 |
| 288 | N | NADGCPSMTV | 13 |
| 289 | E | EDTQGANSVHYRW | 15 |
| 290 | C | CLMYVEKAIFQTDSRNHG | 37 |
| 291 | D | DSE | 2 |
| 292 | P | PVGICLFTAMYS | 11 |
| 293 | L | LFYVAIMS | 5 |
| 294 | R | RKYLSAVIMCHFTQE | 14 |
| 295 | D | DSQTGNHRAFLWECIVPM | 9 |
| 296 | E | EDQGSPVHWLI | 7 |
| 297 | G | GCSDANTVIQ | 14 |
| 298 | I | IVLEMRTQFDYKHASGN | 42 |
| 299 | D | NDAELQGVFTKSHIRMC | 61 |
| 300 | F | FYHLMVI | 11 |
| 301 | Y | YCFVALGHSITEQK | 16 |
| 302 | R | RHGTMDKLASQEVNYFC | 69 |
| 303 | R | LRKQNETADSVHMYF | 35 |
| 304 | L | LFIMCAV | 5 |
| 305 | M | IMLQVASDCTKREYWGNFH | 76 |
| 306 | A | EARNSKQHIDLGCTMV | 77 |
| 307 | A | ANLSDGCTHQVEFR | 17 |
| 308 | G | GDHNRLKASQE | 5 |
| 309 | V | VNISGCKL.T | 13 |
| 310 | P | EPASNLQKRGDTVI.HY | 79 |
| 311 | A | ASTVCLR.INF | 24 |
| 312 | R | QRKHVCTINSDEYA.LFM | 63 |
| 313 | C | CGASFLIEVT.MHQDYWRN | 48 |
| 314 | R | RLEHSKQDINFTVPCY.AMW | 62 |
| 315 | Q | QVMTLIERSCHPK.AGNYDF | 50 |
| 316 | V | VARNFYWLHEIT.GMQC | 22 |
| 317 | M | MLASNHIKQGPED.VRFT | 50 |
| 318 | G | GADEN.HSQTRK | 9 |
| 319 | T | TMSHIVYLGCQA.FN | 36 |
| 320 | C | ICAMTVGSFPYWLD.HNK | 26 |
| 321 | H | HP.V | 1 |
| 322 | A | GATCSLVWI.FDPNME | 18 |
| 323 | G | TGNACSYIFWH.VML | 14 |
| 324 | D | EDSGMAIFQYN.TRHPLWVC | 46 |
| 325 | M | IMVLNS.RTFYWQGHADECKP | 55 |
| 326 | F | FLMIQASGTVKNHD.YWRE | 35 |
| 327 | V | .VAPGTRMSYQHEDLINFW | 50 |
| 328 | A | LAVISQGKDRHTENMP.W | 74 |
| 329 | V | GVAFTYLKN.SIHPWEDMQR | 66 |
| 330 | I | CIMVFL.NTASRYQH | 50 |
| 331 | P | MPTAS.QDNKEHRGY | 42 |
| 332 | D | NDEA.QMSPVYTILFCRKGH | 50 |
| 333 | V | VILEMAHF.QSRKTN | 78 |
| 334 | S | SFYVHNAIGCTKML.WPE | 37 |
| 335 | A | QARLHFDKSNMEWIYVCGT. | 74 |
| 336 | D | EDVMANTGRKILQS.HFPY | 80 |
| 337 | T | TLSAGVQIFCHYMENW.RD | 31 |
| 338 | A | ALVIMTNKFRSQCDEHY.W | 73 |
| 339 | A | ARDGKSLENVQTHM.IYW | 84 |
| 340 | D | SDMNELK.AFRQGVTPHIWY | 57 |
| 341 | I | IMLVRT.DQFYAKWESCGN | 51 |
| 342 | A | ARSGKVTC.LEDIFYMQHNW | 74 |
| 343 | R | NRSGAVQE.ILKHYDTWM | 86 |
| 344 | T | FTWERI.GAVHYNSLDCQMK | 44 |
| 345 | A | CALVTI.MYFWS | 38 |
| 346 | K | RKTADWYQEN.SIGCHMLV | 76 |
| 347 | G | HGELRASQNK.WDVMTYFP | 82 |
| 348 | G | .GVTCLRAQMIFHYNEKSPD | 72 |
